# Lineage tracing reveals photoreceptor precursor cell subpopulations that contribute to murine retinogenesis

**DOI:** 10.3389/fcell.2026.1814134

**Published:** 2026-06-04

**Authors:** Joseph J. Yano, Zhangyong Wei, Krishna J. Gajjar, Kaidi T. Barati-Stec, Emma Yang, Katherine E. Uyhazi

**Affiliations:** Department of Ophthalmology, Scheie Eye Institute, University of Pennsylvania Perelman School of Medicine, Philadelphia, PA, United States

**Keywords:** lineage tracing, neuroblast, photoreceptor, retinal development, retinal organoid, single cell RNA-sequencing

## Abstract

**Introduction:**

Cell-based therapies hold great promise for treating late-stage retinal degenerative diseases. However, photoreceptor cell transplantation has been limited by poor cellular integration, suggesting that an ideal population of donor cells has not yet been defined.

**Methods:**

Here, we utilized single-cell RNA-sequencing and lineage tracing to assess the heterogeneity of photoreceptor precursor cells during murine retinal development.

**Results:**

Subgroup analysis revealed three transcriptionally distinct populations of *Crx* + cells from postnatal day 2–6 retinas which are consistent with early (*Dll1*+), intermediate (*Neurod4*+), and late (*Prom1*+) photoreceptor precursor cells. Lineage tracing with subpopulation-enriched Cre mice showed that *Dll1*+, *Neurod4*+, and *Prom1*+ cells all generate photoreceptor cells, and captured dynamic changes in gene expression suggestive of sequential states of photoreceptor differentiation. Transcriptomic analysis revealed that similar *CRX* + subpopulations are present in maturing human retinal organoids.

**Conclusion:**

These findings highlight the heterogeneity of neonatal photoreceptor precursor cells, and could help inform future strategies to isolate an optimal population of cells for retinal regeneration.

## Introduction

Retinal degeneration is a leading cause of blindness, affecting over two million people worldwide (Berger et al., 2010; Flaxman et al., 2017; Ludwig and Gamm, 2021). Vision loss is caused by the death of photoreceptor cells, the light sensitive neurons in the retina that perform phototransduction. Because mammalian photoreceptors do not regenerate, loss of these cells results in irreversible blindness. Cell-based therapies, in which healthy photoreceptor cells are transplanted into regions of retinal atrophy, are therefore an attractive approach to replace photoreceptors and restore visual function regardless of the underlying cause of disease. Numerous studies have demonstrated that donor- or induced pluripotent stem cell (iPSC)-derived photoreceptor precursor cells transplanted as dissociated cells (MacLaren et al., 2006; Pearson et al., 2012; Singh et al., 2013; Gasparini et al., 2022), sheets of cells (Seiler and Aramant, 2012), or cells seeded on bioscaffolds (Han et al., 2022) can survive in the subretinal space, form synaptic connections with host bipolar and horizontal cells, and result in detectable improvements in visual function (Santos-Ferreira et al., 2016b; Lakowski et al., 2011; West et al., 2009; Aghaizu et al., 2017; Warre-Cornish et al., 2014; Eberle et al., 2011; Bartsch et al., 2008). However, progress in this field has been hindered by low cellular integration rates (<1%) and poor functional recovery (Eberle et al., 2011; Lakowski et al., 2011; MacLaren et al., 2006; West et al., 2009; Bartsch et al., 2008). In addition, many transplanted cells remain at the injection site as a subretinal mass; these unintegrated cells could impede the diffusion of choroidal oxygen and nutrients, or even lead to worsening atrophy and vision loss.

While there are many factors that undoubtedly affect the integration of transplanted cells - including the host environment, stage of disease, and immune response - one of the most critical factors is the identity of the donor cell itself. Venugopalan *et al.* have elegantly shown that neuronal cells that survive well *in vitro* also have the highest rates of *in vivo* transplantation survival (Venugopalan et al., 2016), suggesting that intrinsic characteristics of donor cells can directly impact the success of transplantation. Photoreceptor precursor donor cells are commonly selected using photoreceptor specific surface markers (CD73, CD133) (Eberle et al., 2011; Lakowski et al., 2015; Lakowski et al., 2011; Lakowski et al., 2018), or transgenic mice or cell lines (*Nrl*- or *Crx-eGFP*) (Lakowski et al., 2011; MacLaren et al., 2006; West et al., 2010). However, these markers are expressed in both developing and mature photoreceptors and thus lack developmental specificity (Furukawa et al., 1997; Morrow et al., 2005; Furukawa et al., 2002). Prior transplantation studies have demonstrated that embryonic progenitor cells and mature photoreceptor cells have poor survival and integration potential compared to donor cells isolated from early (P2-P6) postnatal retinas (MacLaren et al., 2006; Pearson et al., 2012; Gust and Reh, 2011; Singh et al., 2013; Aghaizu et al., 2017), but beyond the gross age of donor retinas, few studies have attempted to further define subpopulations of photoreceptor precursor cells for retinal regeneration (Lakowski et al., 2015; Lakowski et al., 2011; Lakowski et al., 2018; Bai et al., 2024). Additionally, much of the literature defining this time window is confounded by cytoplasmic material transfer (CMT) (Pearson et al., 2016; Santos-Ferreira et al., 2016a; Singh et al., 2016; Ortin-Martinez et al., 2017), in which donor cells form connections with recipient photoreceptor cells and exchange mRNAs, organelles, and proteins, including fluorescent labels (Kalargyrou et al., 2021; Ortin-Martinez et al., 2021). Taken together, these findings suggest that an optimal population of donor cells with robust integration potential has not yet been defined, and that a better understanding of photoreceptor development may improve the efficacy of cell replacement strategies.

**FIGURE 1:**
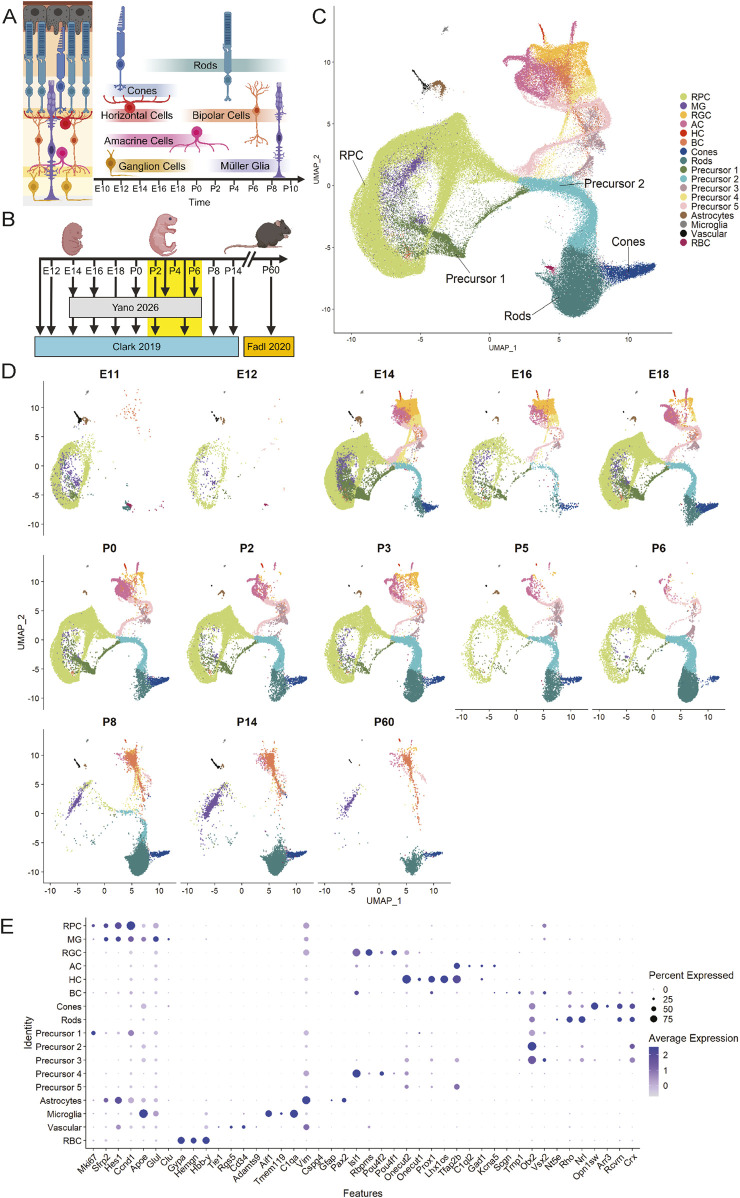
Generating a single-cell RNA-sequencing dataset of murine retinal development. **(A)** Diagram of the retinal layers, cell types, and developmental birth order. Generated in BioRender. **(B)** Schematic of ages used to generate primary scRNA-seq data (Yano 2026), which were integrated with published scRNA-seq data (Clark et al., 2019; Fadl et al., 2020). Generated in BioRender. **(C,D)** UMAP of E11-P60 mouse retinal development data annotated by cell identity **(C)** and faceted by sample age **(D)**. **(E)** Dot plot of differentially expressed genes used to assign cluster identities in E11-P60 mouse retinal developmental data. RPC, retinal progenitor cells; MG, Müller glia; RGC, retinal ganglion cells; AC, amacrine cells; HC, horizontal cells; BC, bipolar cells.

Photoreceptors, along with the other major retinal cell types (retinal ganglion, amacrine, horizontal, bipolar, and Müller glia cells) are derived from a common pool of retinal progenitor cells (RPCs) (Holt et al., 1988; Wetts and Fraser, 1988; Turner and Cepko, 1987) and develop in a highly stereotyped and conserved birth order (Figure 1A) (Belecky-Adams et al., 1996; Young, 1985). Once retinal progenitor cells complete their terminal division, the nascent post-mitotic precursor cells become specified to their terminal cell fates. Photoreceptor precursor cells are marked first by the expression of the transcription factors *Otx2* (Nishida et al., 2003; Koike et al., 2007) and *Crx* (Furukawa et al., 2002; Furukawa et al., 1997), and foundational work has revealed the genes necessary for photoreceptor differentiation, maturation, and function including *Nrl* (Kumar et al., 1996; Rehemtulla et al., 1996; Mears et al., 2001), *Rho* (Humphries et al., 1997), and *Nr2e3* (Bumsted O'Brien et al., 2004; Cheng et al., 2004). In rodents, nascent photoreceptor precursor cells can take up to 2 weeks to initiate *rhodopsin* expression (Morrow et al., 1998), during which distinct developmental stages have been described (Rapaport et al., 2004), though it is unclear if these represent progressive stages of photoreceptor precursors with functional differences. Additionally, since development occurs in temporal and spatial waves across the retina (Carter-Dawson and LaVail, 1979; Masland, 2001; Bassett and Wallace, 2012; Venters et al., 2013; Venters et al., 2015; Aavani et al., 2017), a single timepoint likely contains a heterogenous population of photoreceptor precursor cells at distinct stages of maturation that cannot be resolved by traditional markers. Such cellular heterogeneity has been shown to directly influence the differentiation potential in other stem cell systems (Zhaosong et al., 2021; Kubo et al., 2005), and underscores the need to more precisely define the developmental dynamics of photoreceptor precursor cells.

Single cell RNA sequencing (scRNA-seq) has been successfully used to advance our understanding of the cellular heterogeneity of retinal bipolar (Shekhar et al., 2016), ganglion (Laboissonniere et al., 2017), and foveal versus peripheral retinal cells (Peng et al., 2019). Recently, scRNA-seq analysis of fetal human retinal samples identified sub-states of immature photoreceptor precursors predisposed to form retinoblastoma tumors (Shayler et al., 2025), but thus far has not been extensively applied to understanding the diversity of maturing photoreceptor precursors. In this study, we utilized scRNA-seq and genetic lineage tracing to identify subpopulations of *Crx* + photoreceptor precursor cells in the neonatal mouse retina. These transcriptionally distinct cell populations contribute to photoreceptor cell development, and have comparable populations in human retinal organoids. Together, these findings reveal the heterogeneity of photoreceptor precursor cells in the developing retina which may help guide future retinal regenerative strategies.

## Materials and methods

### Mice

*Dll1-GFP-IRES-Cre-ERT2* (van Es et al., 2012) mice were generated by Dr. Hans Clevers (Hubrecht Institute, Netherlands) and obtained from Dr. Yibin Kang (Princeton University). *Neurod4-CreERT2* (Hirata et al., 2021) mice were cryorecovered from the Riken Institute (CDB0511T). *Prom1-CreERT2* (Zhu et al., 2009) mice were cryorecovered from The Jackson Laboratory (B6N; 129S-*Prom1* ^*tm1 (cre/ERT2)Gilb*^/J; strain 017743). Ai9 (B6.Cg-*Gt (ROSA)26Sor* ^*tm9 (CAG-tdTomato)Hze*^/J, strain 007909) (Madisen et al., 2010) and WT C57BL/6J (strain 000664) mice were obtained from The Jackson Laboratory. For lineage tracing experiments, Cre recombination was induced with a single oral delivery of tamoxifen (Butchbach et al., 2007; Kaiser and Feng, 2019) (50 mg/kg body weight) using flexible gavage needles (FTP-20-38-50, Instech Labs Inc) given to pups at P1 or P2. Tamoxifen free base (T5648, Sigma-Aldrich) was prepared at a stock concentration of 20 mg/mL in corn oil (C8267, Sigma). Eyes were harvested at P4, P6, P8 and P30 as indicated. Genotyping was confirmed prior to all experiments, and both male and female mice were used in these studies. Genotyping primers used in this study are listed in Supplementary Table S1. All animals were bred and maintained in the animal facilities of The University of Pennsylvania under a 12-h light/12-h dark cycle with free access to food and water.

### Retinal dissociation

Eyes were enucleated and retinas were dissected in cold 1xDPBS (all buffers used in this study lacked Ca^2+^ and Mg^2+^ unless specifically noted otherwise). For single-cell RNA-sequencing, four to eight retinas from C57BL/6J mice were pooled for each timepoint. For flow cytometry analysis, one to two retinas were used for each sample and timepoint. Retinas were enzymatically digested in papain (0.6 mg/mL papain, LS003119, Worthington; 0.2 mg/mL L-Cysteine, C-7352, Sigma; 0.02 mg/mL DNase I, D4527, Sigma; 1xDPBS) at 37 °C for up to 10 min. Papain digestion was stopped by Lo-Ovo trypsin inactivator solution (1.5 mg/mL, LS003587, Worthington; 1.5 mg/mL BSA; 0.02 mg/mL DNase I, D4527, Sigma; 1xDPBS, pH 7.4). Retinas were then mechanically digested by gentle trituration and additional DNase I was added as needed to minimize tissue clumping. Dissociated cells were then filtered through a 40 μm cell strainer to remove aggregates and pelleted by centrifugation at 100 *g* for 10 min at room temperature.

### Fluorescence-activated cell sorting (FACS)

For flow and qPCR analysis of neonatal lineage traced cells, dissociated retinal cells from Cre + pups were resuspended in FACS buffer (1% BSA, 1xHBSS) and labeled with DAPI (0.5 ug/mL), CD133-AF647 (1:100, AB_2566013, BioLegend), and CD73-PE/Cy7 (1:100, AB_2716103, BioLegend), and analyzed and sorted on a BD FACS Symphony S6 Sorter (BD Bioscience). Cells from WT littermates were labeled with DAPI only and used for compensation, along with AbC Total Antibody Compensation beads (A10513, Invitrogen) labeled with each antibody. tdTomato compensation beads were prepared by conjugating beads with anti-His antibody (1:500, AB_914704, GenScript) and His-tagged recombinant tdTomato protein (1 μg, TP790045, OriGene). GFP BrightComp eBeads Compensation Bead (A10514, Invitrogen) were also used for the *Dll1-Cre;Ai9* sample analysis to account for the EGFP reporter. Samples were gated on forward and side scatter area, height, and width to remove debris and doublets, and DAPI to remove dead cells. For downstream qPCR analysis, 50,000-200,000 tdTomato + cells were isolated for each sample and timepoint. For flow analysis, 10,000 tdTomato + cell events were recorded, and data were processed using FlowJo (version 10.10.0). Full minus one (FMO) controls were used to set up Prom1-AF647 and CD73-PE/Cy7 negative gates.

### Single cell RNA sequencing

Dissociated retinal cells were purified by FACS prior to sample submission. Cell pellets were resuspended in FACS buffer, labeled with DAPI (0.5 μg/mL), and analyzed and sorted on a BD FACS Aria Cell Sorter (BD Bioscience). Samples were gated on forward and side scatter area, height, and width to remove debris and doublets, and DAPI to remove dead cells. Following FACS purification, sorted cells were manually counted using Trypan Blue (25-900-CI, Corning) and a hemocytometer. Approximately 20,000 live cells were resuspended at 700–1220 cells/µL (0.04% BSA, 1xDPBS) and submitted to the Center for Applied Genomics (CAG) Core at the Children’s Hospital of Philadelphia (CHOP) for library preparation and single-cell RNA-sequencing using the 10x Genomics platform, and preliminary CellRanger processing. ScRNA-seq analysis was performed in RStudio (build 2024.04.2 + 764, R version 4.1.2) using the Seurat package (Hao et al., 2021) (version 4.3.0). Dying cells were filtered out by excluding cells with >15% mt-RNA. Additional filtering was performed to remove empty cells and potential doublets (300 < nFeature_RNA <4000). The clusters with the highest expression of *Crx* were identified and used for subset analysis of photoreceptor cells. Clustering for subset analysis used resolutions of 0.2 (mouse *Crx +* P2-P6 subset) and 0.25 (human organoid *CRX +* subset). Pseudotime analysis was performed using the Monocle3 package (Cao et al., 2019; Qiu et al., 2017a; Qiu et al., 2017b; Trapnell et al., 2014) (version 1.2.9) and *Ccnd1* or *Olig2* (Hafler et al., 2012) as the reference gene. Previously published scRNA-seq datasets for mouse and human retinal organoids were accessed from GSE118614 (Clark et al., 2019), GSE153674 (Fadl et al., 2020), GSE142526 (Sridhar et al., 2020), and GSE235577 (Dorgau et al., 2024). Primary scRNA-seq data generated in this study have been deposited at the National Center for Biotechnology Information (NCBI) Gene Expression Omnibus (GEO) at GEO accession number GSE297036.

### RNA isolation & qPCR analysis

For qPCR analysis of developing mouse retinas, eyes were harvested from WT mice at E14, E16, P0, P3, P4, P5, P6, P15, and P30. Two retinas from C57BL/6J mice were pooled for each timepoint. Following enucleation, retinas were isolated from the RPE and choroid, collected into 1 mL of TRIZOL reagent (15596026, Thermo Fisher), and gently digested with a Dounce homogenizer. For FACS isolated samples, cell pellets were resuspended in 1 mL of TRIZOL reagent, and gently digested by trituration. RNA was isolated following manufacturer’s instructions. cDNA synthesis was performed using the High-Capacity cDNA Reverse Transcription Kit (4374966, Applied Biosystems). Primers were designed using the NCBI Primer BLAST tool (Ye et al., 2012). qPCR was performed in triplicate using SybrGreen reporter following manufacturer’s instructions (A25741, Applied Biosystems). Relative average fold changes were calculated using ΔΔCt analysis, using *B2m* as the housekeeping gene. E14 and P4 samples were used as the references for the developing mouse retina analysis and FACS isolated samples, respectively. The qPCR primers used in this study are listed in Supplementary Table S2.

### Histology and immunofluorescence

Following enucleation, the cornea and lens were removed, and eye cups were fixed in paraformaldehyde (4%, 1xDPBS) for 1-2 h at room temperature or overnight at 4 °C. Eye cups were then placed into sucrose solution (30%, 1xDPBS) at 4 °C overnight, embedded in Tissue-Tek OCT freezing media (VWR), and processed on a cryostat (Leica) to prepare 12 µm sections. Slides were air dried and stored at −20 °C prior to immunofluorescence analysis. Sections were first rehydrated with 1xDPBS (x3 5-min washes), and then blocked in IF permeabilization buffer (5% horse serum, 1% goat serum, 3% BSA, 0.01% TritonX-100, 1xDPBS) for 1 h at room temperature. Sections were then washed in PBT buffer (0.1%Tween-20, 1xDPBS; x3 5-min washes), and incubated in primary antibodies diluted in IF buffer (5% horse serum, 2% goat serum, 3% BSA, 1xDPBS) overnight at 4 °C. Following 1xPBT washes (x3 5-min washes), sections were incubated in secondary antibody diluted in IF buffer for 1 hour at room temperature. Sections were washed again with 1xPBT (x3 5-min washes) and then counterstained with Hoechst-33342 (2.5 μg/mL, 1xDPBS) for 10 min at room temperature. Retinal sections were washed with 1xDPBS (x2 5-min washes) and covered with ProLong Gold Antifade Mountant (P36930, Invitrogen), and cover slips were sealed with nail polish. The following primary antibodies were used: anti-RFP (1:250, AB_2209751, Rockland), anti-CD73 (1:350, AB_1089066, BioLegend), anti-Rhodopsin (1:500, AB_10696805, abcam), anti-Cone Arrestin (1:500, AB_1163387, Millipore), anti-Pax6 (1:2000, AB_2565003, BioLegend), anti-Vsx2 (1:50, AB_2216010, Invitrogen), anti-Glutamine Synthetase (1:600, AB_1950421, GeneTex). The following secondary antibodies were used: Alexa Fluor 488 Goat anti-Rabbit (1:500, AB_2576217, Invitrogen), Alexa Fluor 647 Goat anti-Rabbit (1:500, AB_2535813, Invitrogen), Alexa Fluor 750 Goat anti-Rabbit (1:500, AB_2535710, Invitrogen), Alexa Fluor 647 Goat anti-Rat (1:500, AB_141778, Invitrogen), CF 488A Donkey anti-Sheep (1:500, AB_10583179, Biotium). Slides were imaged with a Zeiss 980 confocal microscope. For consistency, images were captured from central retinal sections proximal to the optic nerve. When necessary, images were taken from more peripheral areas to capture examples of rare bipolar and Müller glia cell labeling in the *Neurod4-Cre +* or *Prom1-Cre +* linage tracing samples. Images were processed and quantified in FIJI (ImageJ, version 1.54f) (Schindelin et al., 2012).

### RNA fluorescent *in situ* hybridization (RNA-FISH)

RNA-FISH was performed on 12 µm frozen retinal sections using the Advanced Cell Diagnostics RNAscope Multiplex Fluorescent Reagent Kit v2 (323100, Bio-Techne). Manufacturers’ instructions were followed with the following exception: target retrieval was performed manually, using 700 mL of 1x Target Retrieval Reagent (5 min at 100 °C). Probe signals were developed using Opal 520 (1:2000, FP1487001KT, Akoya BioSciences), Opal 570 (1:1500, FP1488001KT, Akoya BioSciences), and TSAVivid 650 (1:1500, 323273, Bio-Techne) reporters diluted in TSA Buffer.

### Quantification and statistical analysis

Cell counts and quantification for the P30 lineage tracing samples were performed using maximum intensity projection images and the multipoint tool in FIJI. Cell counts were taken from a 353.33 µm^2^ area. The total number of tdTomato + cells was counted from 3-4 sections from 5-7 eyes per Cre line. The number of tdTomato + cells coexpressing INL cell markers was counted from 3-5 sections from 3-5 eyes. For counting the number of tdTomato + cells in the ONL of *Neurod4-Cre +* samples, a cropped image 1/10th the width of the imaged ONL was used and multiplied by 10 to estimate the total number of tdTomato + ONL cells. The number of tdTomato + cells in the ONL or INL was then divided by the total number of tdTomato + cells per image to calculate the proportion of labeled cells in each retinal layer. The number of tdTomato + cells coexpressing INL cell markers was divided by the estimated average number of tdTomato + INL cells to calculate the proportions of labeled amacrine, bipolar or Müller glia cells. To account for any inter-eye effects from the left and right eyes of our samples, we utilized generalized estimating equations (GEE) analysis to calculate the standard error of the means (SEM) (Ying et al., 2017). All datasets were approximately normal (as visualized by Q-Q plots) except for the counts of Müller glia cells, which were analyzed with Poisson distribution. The proportions of labeled ONL and INL cells were analyzed by Least Squares Means (LSM) with Bonferroni post-hoc test (α = 0.05) for multiple comparisons. GEE and LSM analysis was performed in SAS Studio (version 3.81). For the flow cytometry analysis of the FACS isolated lineage tracing time course samples, proportions of Prom1+/CD73+ cells were assessed with one-way ANOVA with Tukey’s post-hoc test (α = 0.05) for multiple comparisons. For the qPCR analysis of the FACS isolated lineage tracing time course samples, gene expression changes were assessed with one-way ANOVA of the ΔCt values with post-hoc Dunnett’s test (α = 0.05) using the P4 timepoint as the reference to perform multiple comparisons. Data were compiled and graphed using GraphPad Prism version 10.4.1 for Windows (GraphPad Software, Inc; La Jolla, CA).

## RESULTS

### Generating a single-cell RNA-Sequencing dataset of murine retinal development

To better understand the fundamental heterogeneity of photoreceptor cells during normal development (Figure 1A), we characterized the transcriptional profile of the mouse retina at E14, E16, P0, P3, and P6 using scRNA-seq (Figure 1B; Supplementary Figure S1A). Given the relatively rapid nature of transcriptional changes that can occur during development, we selected these timepoints to complement existing scRNA-seq datasets during the early postnatal period, a critical window for rod photoreceptor development. By integrating our scRNA-seq data with additional timepoints from prior studies (Clark et al., 2019), we developed a time resolved atlas of transcriptional changes every 24-48 h during this important developmental window that has been increasingly used as a source of cells for transplantation (Figure 1B, yellow box). Additionally, we incorporated data from adult (P60) retinas to include signatures from fully matured cells (Fadl et al., 2020) (Figure 1B; Supplementary Figure S1B). ScRNA-seq data were integrated for analysis (Figure 1C) (Hao et al., 2021), faceted by sample age (Figure 1D), by data source (Supplementary Figure S1B) and by cell cycle regression analysis (Supplementary Figure S1C). Panels of retinal cell type marker genes were used to assign cluster identities (Figure 1E). Retinal progenitor cells were most abundant in the early timepoints, and appeared along with early-born retinal ganglion, horizontal, and cone photoreceptor cells. Diminishing numbers of progenitor cells and increasing numbers of late-born bipolar, Müller glia, and rod photoreceptor cells were detectable in postnatal retinas (Figure 1D). In addition to the retinal progenitor and mature retinal cells, we also identified several intervening precursor populations based on marker gene expression and adjacent clusters (Figure 1C). Precursor 2 (*Otx2*+/*Crx*+) and Precursor 3 (*Otx2*+/*Vsx2*+) are photoreceptor and bipolar precursors, respectively, with Precursor 1 (*Otx2*+) potentially shared photoreceptor/bipolar precursors. Precursor 4 (*Pou4f2*+) and Precursor 5 (*Tfap2b*+) appear to be ganglion and amacrine precursors, respectively. These trajectories were further corroborated by pseudotime analysis (Supplementary Figure S1D) (Trapnell et al., 2014; Qiu et al., 2017a; Qiu et al., 2017b; Cao et al., 2019). All major retinal cell types were captured and demonstrated expected developmental trajectories.

### Subgroup analysis of *Crx* + cells in P2-P6 retinas reveals distinct subpopulations of photoreceptor precursor cells

**FIGURE 2:**
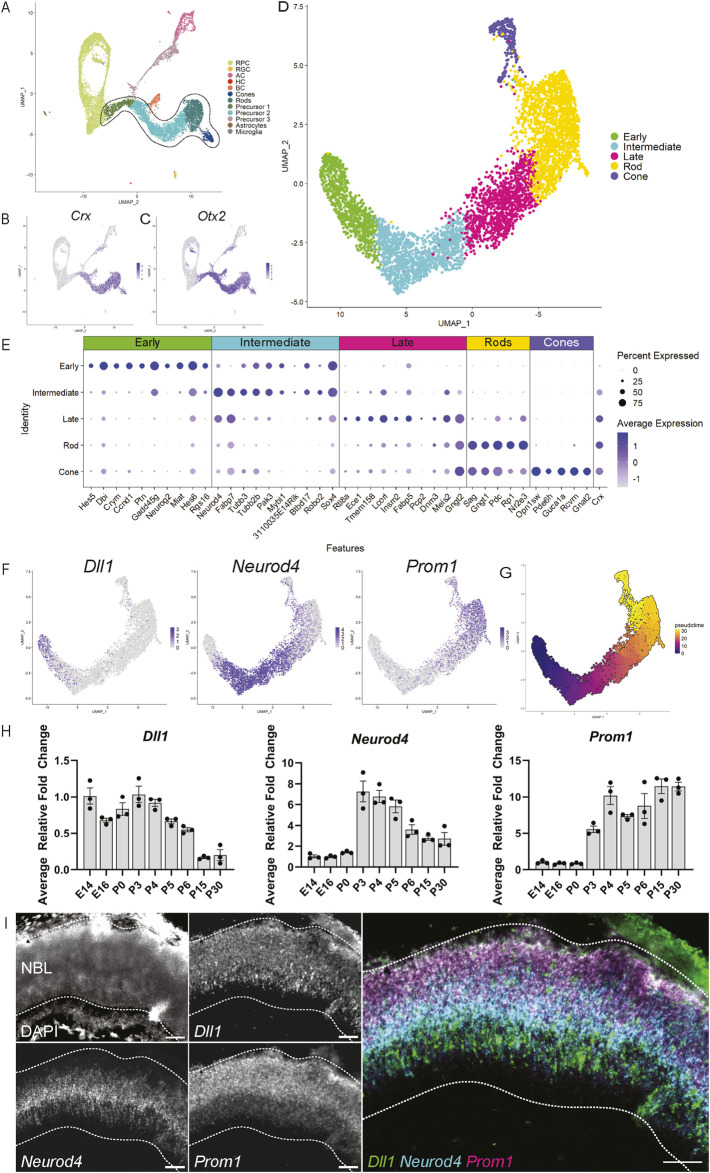
Subgroup analysis of *Crx* + cells in P2-P6 mouse retinas reveals distinct subpopulations of photoreceptor precursor cells. **(A–C)** UMAP of P2-P6 mouse retina annotated with cell identities **(A)**; *Crx* **(B)** and *Otx2* expression **(C)** was used to identify and further subset developing photoreceptor precursors (dashed outline). **(D)** Subcluster analysis of *Crx +* cells reveals mature rods (yellow) and cones (purple), as well as several distinct clusters of photoreceptor precursor cells (green, cyan, magenta). **(E)** Dot plot of top differentially expressed genes used to assign P2-P6 *Crx +* subpopulation identities. **(F)** UMAP of P2-P6 *Crx* + clusters enriched for representative markers of the early (*Dll1+*), intermediate (*Neurod4+*), and late (*Prom1+*) photoreceptor precursor subpopulations. **(G)** Pseudotime trajectory analysis of *Crx +* subsets performed with Monocle3, using *Olig2* as a reference. **(H)** Average fold enrichment of representative marker genes in E14-P30 retinas by qPCR, relative to E14 samples. Data represented as mean ± SEM. **(I)** RNA-FISH analysis reveals distinct spatial expression of *Dll1* (green), *Neurod4* (cyan), and *Prom1* (magenta) in the P4 retina. DAPI nuclei stain used to define the boundaries of the neuroblast layer (NBL, dashed outlines); outer retina is oriented towards the top of the image. Scale bars = 10 µm.

Prior studies of photoreceptor precursor transplants have reported that cells collected from early postnatal retinas (P2-P6) have the highest rates of donor cell survival and integration compared to cells isolated from earlier or later timepoints (MacLaren et al., 2006; Pearson et al., 2012; Aghaizu et al., 2017; Gust and Reh, 2011; Singh et al., 2013), though these reports warrant reevaluation due to cytoplasmic material transfer (Pearson et al., 2016; Santos-Ferreira et al., 2016a; Singh et al., 2016; Ortin-Martinez et al., 2017). This time period coincides with the peak of rod photoreceptor genesis, and an enrichment of precursor cells expressing *cone-rod homeobox* (*Crx*), a transcription factor required during retinal development for photoreceptor specification and maturation (Furukawa et al., 1997) (Figure 1E; Supplementary Figure S1E). To investigate the transcriptional profile of developing photoreceptor cells during this stage, we performed a subgroup analysis of the early postnatal period (P2-P6) (Figure 2A). We identified the trajectory of photoreceptor cell development by identifying clusters with strong *Crx* and *Otx2* expression (Figures 2B,C). Subgroup analysis of these cells in P2-P6 retinas (Figure 2A, dashed outline) revealed five clusters (Figure 2D). Based on differentially expressed marker genes (Figures 2E,F) and pseudotime analysis (Figure 2G), these include mature cone (purple) and rod (yellow) photoreceptor cells, as well as three clusters that correspond to early (green), intermediate (cyan), and late (magenta) stage photoreceptor precursor cells (Figure 2D). Cell cycle regression analysis showed that all clusters had low S and G2M scores, suggesting that these are post-mitotic cells (Supplementary Figure S2A). The earliest group (green) is enriched in genes associated with neural specification (*Neurog2*, *Ascl1*, *Pax6*) (Figure 2E; Supplementary Table S3), suggesting that these are precursor cells that have recently exited the cell cycle and begun differentiating. The intermediate group (cyan) is marked by genes associated with photoreceptor development (*Neurod4*, *Abca4*, *Sox4*), and cytoskeletal dynamics and axonal positioning (*Tubb3*, *Robo2*, *Stmn2*) (Figure 2E; Supplementary Table S4). The late group (magenta) is associated with late-stage precursors and maturing photoreceptor markers (*Prom1*, *Nrl*, *Nr2e3*) (Figure 2E; Supplementary Table S5).

We then identified candidate marker genes enriched in the early (*Dll1+)*, intermediate (*Neurod4+)*, and late (*Prom1+)* groups (Figure 2F). Gene expression was validated by qPCR of retinal lysates from E14-P30 mice (Figure 2H; Supplementary Table S6), and by RNA-FISH of retinal sections from P4 mice (Figure 2I). Interestingly, these markers have distinct spatial expression patterns in the developing neuroblast layer (dashed lines in Figure 2I). *Dll1* (a marker of the early group) is expressed throughout the neuroblast layer in the P4 retina, *Neurod4* (a marker of the intermediate group) is expressed in the medial neuroblast layer, and *Prom1* (a marker of the late group) is expressed most strongly in the apical neuroblast (Figure 2I). Importantly, we observed expression of all three subpopulation markers in the outer neuroblast, consistent with *Crx* expression (Supplementary Figure S2B) and the location of the presumptive outer nuclear layer where mature photoreceptors ultimately reside. These data suggest that photoreceptor precursor cells are heterogeneous, and consist of several transcriptionally distinct subpopulations that coexist in the retina at the same developmental age.

### *Dll1+*, *Neurod4+*, and *Prom1+* cells generate mature photoreceptors

**FIGURE 3:**
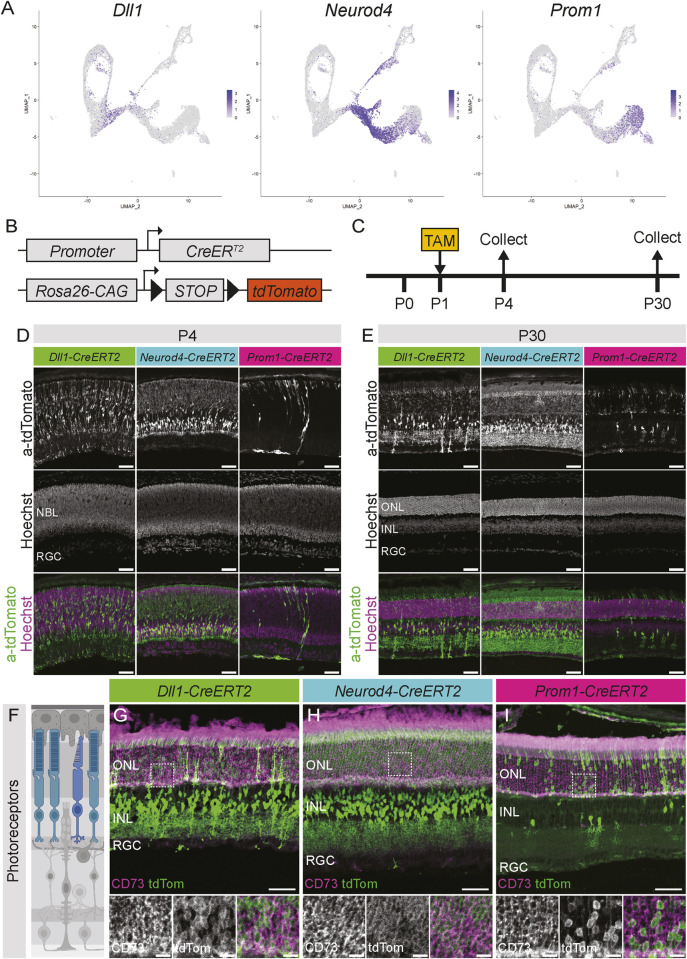
*Dll1+*, *Neurod4+*, and *Prom1+* cells generate mature photoreceptors. **(A)** UMAPs of *Dll1*, *Neurod4*, and *Prom1* expression in P2-P6 mouse retinas. **(B)** Subpopulation enriched Cre mice (upper panel) were crossed with Ai9 reporter mice (lower panel). **(C)** Lineage tracing experiments were performed by administering tamoxifen (TAM) to P1 or P2 pups to permanently label cells with tdTomato; eyes were harvested at P4 and P30 for histological analysis. **(D,E)** Representative immunofluorescence images of P4 and P30 retinas from *Dll1-CreER;Ai9*, *Neurod4-CreER;Ai9*, and *Prom1-CreER;Ai9* samples stained with anti-tdTomato (green) representing lineage traced cells and Hoechst (magenta) showing nuclear layers. Scale bars = 50 µm. **(F)** Diagram highlighting photoreceptor cell location and morphology in the outer retina. **(G–I)** Representative immunofluorescence images of P30 retinas show co-staining of tdTomato + lineage traced cells (green) with CD73 (photoreceptor cell marker, magenta). Scale bars = 50 µm. Dashed boxes outline areas magnified for cropped insets. Inset scale bars = 10 µm. NBL, neuroblast layer; ONL, outer nuclear layer; INL, inner nuclear layer; RGC, retinal ganglion cell.

To determine if the early (*Dll1*+), intermediate (*Neurod4+*), and late (*Prom1+*) subpopulations of *Crx +* cells identified by our unbiased scRNA-seq analysis contribute to the pool of mature photoreceptors, we performed genetic lineage tracing experiments using inducible Cre reporter lines driven by candidate marker genes. Each promoter was chosen based on a search of candidate genetic markers that were enriched in each population (hence the naming of clusters) (Figure 3A). *Dll1-CreER* (van Es et al., 2012), *Neurod4-CreER* (Hirata et al., 2021), and *Prom1-CreER* (Zhu et al., 2009) mouse lines were used to trace the early, intermediate, and late photoreceptor precursor groups, respectively. Cre mice were crossed with the Ai9 reporter mouse (*Rosa26-CAG-LSL-tdTomato)* (Madisen et al., 2010), so that upon tamoxifen administration, all cells expressing the subpopulation-enriched promoters were permanently labeled with tdTomato (Figure 3B). Tamoxifen was administered orally (Butchbach et al., 2007) at a single dose of 50 mg/kg (Kaiser and Feng, 2019) to one- or two-day old pups to allow for recombination to occur in the neonate retinas. Eyes were then harvested at P4 and P30 for histological analysis (Figure 3C). *Dll1-CreER;Ai9, Neurod4-CreER;Ai9,* and *Prom1-CreER;Ai9* litters that did not receive tamoxifen all had minimal background signal at P4 and P30 timepoints, validating the fidelity of the inducible system (Supplementary Figure S3A,B). Mice treated with tamoxifen at P1 and P2 had normal retinal morphology (Figures 3D,E), suggesting that this tamoxifen dose does not grossly affect retinal development. Cells labeled with the tdTomato reporter were observed in P4 pups from crosses with all three Cre lines. To increase the signal-to-noise ratio of the tdTomato reporter, retinal sections were stained with anti-RFP antibody prior to imaging. The antibody signal strongly coexpressed with the endogenous tdTomato signal (Supplementary Figure S3C,D). Labeled cells in the *Dll1-Cre* + mice were located throughout the neuroblast layer. Labeled *Neurod4-Cre* + cells were found in a striking band medially to basally in the neuroblast layer as well as diffusely in the apical neuroblast layer. Labeled cells in the *Prom1-Cre* + mice were located apically and occasionally spanning the neuroblast layer (Figure 3D). The spatial patterns of these lineage traced cells in the P4 retina are consistent with the expression patterns of marker genes by RNA-FISH at this timepoint (Figure 2I). We next analyzed retinal sections from mice lineage traced to P30 to assess the terminal fates of the labeled cells in adult retinas. tdTomato + cells with photoreceptor cell morphology were present in the outer nuclear layer (ONL) of retinas from all three Cre lines (Figure 3E), and coexpressed with the photoreceptor marker CD73 (Figures 3F–I). The majority of labeled cells were rods based on cellular morphology (long, cylindrical outer segments) and co-staining with rhodopsin (Supplementary Figure S4A,C–E). Cone arrestin appeared to be less frequently expressed in tdTomato + cells compared to rhodopsin (Supplementary Figure S4B,F–H). Together, our lineage tracing experiments suggest that the early, intermediate, and late subpopulations all contribute (albeit not exclusively) to the photoreceptor fate in the mouse retina.

While a majority of labeled cells at P30 were photoreceptors, we also observed labeled cells in the inner nuclear layer (INL) (Figure 3E; Supplementary Figure S5). This is likely due to Cre expression in a small percentage of late RPCs and precursor cells outside of the photoreceptor lineage (Figure 3A). On average, *Prom1-Cre* labeled the greatest number of photoreceptor cells (96.6%) and the fewest non-photoreceptor cells (3.4%) compared to *Dll1*-Cre (76.7% PR/23.3% INL) *and Neurod4*-Cre (88.8% PR/11.2% INL) (Supplementary Figure S5M). The morphology and location of these labeled INL cells were most consistent with amacrine, bipolar, and Müller glia cells (Supplementary Figure S5A–C). We performed additional immunofluorescence analysis of the P30 lineage tracing samples to co-stain with known markers of amacrine cells (Pax6) (Supplementary Figure S5D–F), bipolar cells (Vsx2) (Supplementary Figure S5G–I), and Müller glia cells (Glutamine synthetase [GS]) (Supplementary Figure S5J–L). Of the non-photoreceptor cells, *Dll1-Cre +* retinal sections labeled amacrine, bipolar, and Müller glia cells, while the *Neurod4-Cre+* and *Prom1-Cre +* samples labeled primarily amacrine and bipolar cells with rare Müller cells (0.3% and 4.0%, respectively) (Supplementary Figure S5N). No labeled retinal ganglion cells or horizontal cells were observed. Together, these data suggest that *Dll1*, *Neurod4*, and *Prom1* are enriched but not exclusive to each photoreceptor precursor population, and that all three populations contribute to the mature photoreceptor pool.

### *Dll1+*, *Neurod4+*, and *Prom1+* subpopulations are consistent with sequential photoreceptor precursor states

**FIGURE 4:**
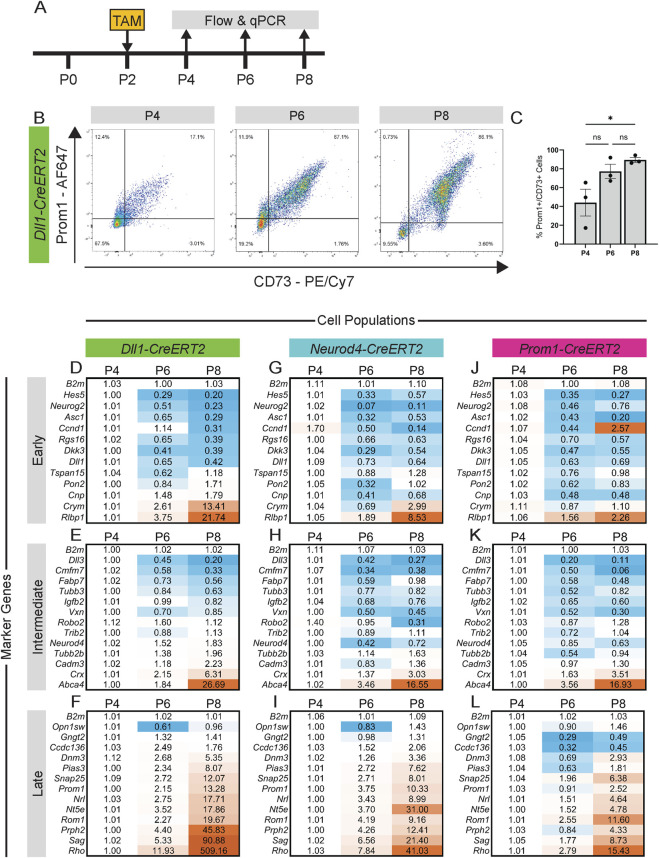
*Dll1+*, *Neurod4+*, and *Prom1+* subpopulations are consistent with sequential photoreceptor precursor states. **(A)** Analysis of lineage traced cells across the neonatal time window was performed by administering tamoxifen (TAM) to P2 pups and harvesting eyes at P4, P6, and P8 for qPCR and flow cytometry analysis. **(B,C)** Flow analysis of Prom1 and CD73 expression in tdTomato+ **(B)** cells for *Dll1-CreERT2* samples at P4, P6 and P8. Quantification of the proportions of Prom1+/CD73+ cells in tdTomato+ **(C)** samples from *Dll1* traced cells. Values are mean ± SEM (n = 3). Statistical test done with one-way ANOVA with Tukey’s post-hoc analysis, *p = 0.0325. **(D–L)** qPCR analysis of tdTomato + cells sorted from P4, P6, and P8 samples. Samples were probed with panels of marker genes for the Early **(D,G,J)**, Intermediate **(E,H,K)**, and Late **(F,I,L)** subpopulations. *B2m* and respective P4 samples were used as references for ΔΔCt analysis. Results are summarized in heat maps displaying average fold changes (downregulated genes indicated in blue; upregulated genes in orange).

We next sought to determine if the early, intermediate, and late subpopulations are sequential stages of photoreceptor precursor maturation as suggested by the pseudotime analysis (Figure 2G), or parallel pathways that can each directly form photoreceptors. To test this, we treated *Dll1-CreER;Ai9, Neurod4-CreER;Ai9,* and *Prom1-CreER;Ai9* litters with tamoxifen at P2, and analyzed tdTomato + cells at P4, P6, and P8 by flow cytometry and qPCR (Figure 4A; Supplementary Figure S6A–F). Cells were stained with antibodies for Prom1 (CD133) (a marker of the late group) and CD73 (a marker of maturing photoreceptors). The proportions of Prom1 and CD73 double positive cells were measured at P4, P6, and P8 from tdTomato + cells (Figures 4B–C; Supplementary Figures S6A–D). *Dll1-Cre +* began with the fewest Prom1+/CD73+ cells at P4 (44.0%), which strongly increased by P6 (77.3%) and P8 (89.4%) (Figures 4B,C; Supplementary Figure S6E), suggesting that Dll1+ cell populations acquire Prom1 expression over time. In contrast, *Prom1-Cre +* samples did not have a significant increase in Prom1+/CD73+ expression from P4 to P8 (Supplementary Figure S6C–E), which would be expected if these cells were already enriched for Prom1 at P4. Interestingly, *Prom1-Cre +* samples showed a steady increase in CD73^+^ expression over time (Supplementary Figure S6C), which is consistent with our qPCR analysis (Figure 4L) and suggests that Prom1 cells are in the later stages of photoreceptor development and maturation, and that Prom1 marks an earlier cell state than CD73.

We then analyzed gene expression changes of representative panels of early, intermediate, and late gene markers in lineage traced cells at P4, P6, and P8 by qPCR (Figure 4D–L; Supplementary Table S7). The *Dll1-Cre +* samples showed the most dynamic gene expression from P4 to P8. Early gene markers were strongly downregulated (Figure 4D, blue boxes), while some intermediate and nearly all late gene markers were strongly upregulated over time (Figures 4E–F, orange boxes), suggesting that permanently labeled “early” population cells may subsequently acquire a signature of the intermediate and late groups. Similarly, the *Neurod4-Cre +* samples had low expression of early gene markers, accompanied by a gradual but strong upregulation of late marker genes from P4 to P8 (Figures 4G–I). Finally, *Prom1-Cre +* cells showed low expression of early and intermediate gene markers at P6 and P8 (Figures 4J–K) and strong upregulation of late gene markers associated with photoreceptor maturation (Figure 4L). Although these experiments were conducted on bulk-sorted tdTomato + cells and therefore cannot resolve the transition states of individual cells, these findings are consistent with–though not definitive evidence for–a sequential model of photoreceptor development in which *Dll1*+, *Neurod4*+, and *Prom1*+ cells progress through distinct developmental states along their trajectory to mature photoreceptors. It would be interesting to employ single-cell-resolution approaches in future studies to more precisely define these transition states.

### *CRX +* photoreceptor precursor subpopulations are present in human retinal organoids

**FIGURE 5:**
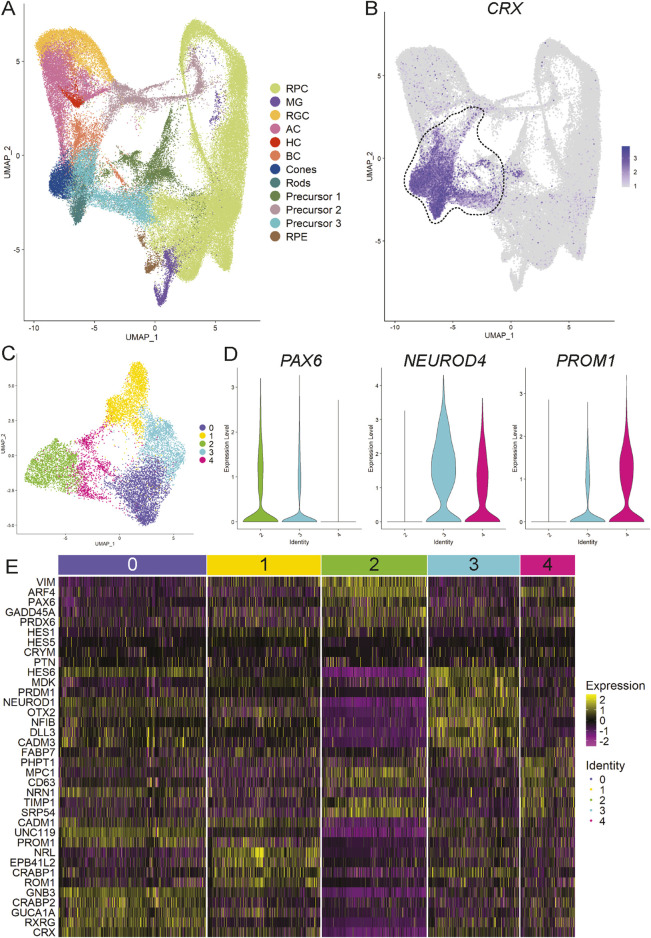
*CRX +* photoreceptor precursor subpopulations are present in human retinal organoids. **(A)** UMAP of integrated human retinal organoid scRNA-seq datasets; clusters annotated by cell identity. **(B)** UMAP of *CRX* expression in human retinal organoids. Dashed outline indicates *CRX* + cells used for subset analysis of developing photoreceptors. **(C)** Subcluster analysis of *CRX* + cells in human retinal organoids reveals distinct clusters reminiscent of the clusters identified in the developing mouse retina. **(D)** Violin plots of *PAX6*, *NEUROD4*, and *PROM1* expression in *CRX* + precursor clusters. **(E)** Heatmap of differentially expressed genes of *CRX* + clusters.

For future therapeutic applications, it would be advantageous to obtain photoreceptor precursor donor cells from cultured sources rather than primary retinal tissues. Human induced pluripotent stem cell (hiPSCs)-derived retinal organoids are three-dimensional structures that recapitulate the development of the human retina, and have become an increasingly popular source of donor cells for photoreceptor transplantation. To investigate the heterogeneity of photoreceptor precursor cells in retinal organoids and to determine if the *Crx +* subpopulations identified in this study are conserved across species, we next compared our mouse data to published scRNA-seq datasets of human retinal organoids (Supplementary Figure S7A,B) (Sridhar et al., 2020; Dorgau et al., 2024). All expected retinal cell types were represented in human retinal organoids (Figure 5A; Supplementary Figure S7C). Subgroup analysis of *CRX +* cells revealed several clusters of maturing human photoreceptor precursor cells (Figures 5B,C; Supplementary Figure S7D). Differentially expressed gene analysis of the clusters identified cone (group 0, purple), and rod (group 1, yellow) photoreceptors (Figures 5C–E). Groups 2 (green) and 4 (magenta) showed enrichment for genes bearing some resemblance to the early and late groups from our mouse scRNA-seq data, respectively. Although expression of *DLL1*, the marker of the early mouse group, was not detected in the human *CRX +* subset, we did observe enrichment of *PAX6*, an additional marker of the early group (Supplementary Table S3) in group 2. Similarly, *PROM1* was enriched in group 4, the presumptive late group (Figure 5D). Interestingly, while neither group had as many differentially expressed genes directly related to photoreceptor development, many genes were related to cellular migration and localization (*MDK*, *FBLN1*, *KIF22*). Lastly, group 3 (cyan) shared many transcriptional signatures with the intermediate group in the developing mouse retina such as *NEUROD4* (Figure 5D,E), and showed strong enrichment for genes related to neurogenesis and axon development (*NEUROD1*, *NFIB*, *ATOH7*, *PRDM1*). Overall, these data suggest that human retinal organoids contain heterogeneous populations of photoreceptor precursor cells that may be evolutionarily conserved across species. Further studies validating the presence of these subpopulations and their utility in cell-based therapies will improve our understanding of retinal development and may refine a suitable source of donor cells for retinal regeneration.

## Discussion

Retinal development is a complex process, with all seven major retinal cell types emerging from a common pool of retinal progenitor cells in overlapping temporal and spatial waves. This results in a heterogeneous pool of progenitor, precursor, and maturing retinal cells. This is especially pertinent to photoreceptors, as many of the genes necessary for photoreceptor precursor cell development, such as *Crx* and *Nrl*, are also required for the maintenance and function of mature photoreceptors cells. While the genes regulating photoreceptor development have been extensively characterized, the developmental heterogeneity of photoreceptor precursor cells is poorly understood, particularly as it relates to isolating an optimal population of cells for regenerative strategies (Lakowski et al., 2011; Lakowski et al., 2015; Lakowski et al., 2018). Recently, there have there been reports of precursor substates in developing fetal retinas (Shayler et al., 2025), as well as brains (Hamed et al., 2022), highlighting the heterogeneity of neural precursor cells. Reducing donor cell heterogeneity results in increased RPE cell engraftment and polarization (Tessmer et al., 2024), enhanced cortical cell survival and cell fate control (Molina-Ruiz et al., 2025; Fortin et al., 2016), and improved cardiac functional recovery (Tripathi et al., 2020; Kawamoto et al., 2006), suggesting that a similar approach may be beneficial in the neural retina.

Previous reports have suggested that donor cells derived from the early neonatal period (P2-P6) exhibit improved integration upon transplantation compared to earlier or later timepoints (MacLaren et al., 2006; Pearson et al., 2012; Singh et al., 2013; Aghaizu et al., 2017; Gust and Reh, 2011), therefore we focused our transcriptomic analysis on this important developmental window. Interestingly, we observed enrichment of *Crx* + precursor cells during this time, with a sharp decline by P8, suggesting that the presence of this population may be important for the survival and functional integration of transplanted cells. Of note, in light of cytoplasmic material transfer that may have confounded these studies, it would be interesting to validate that P2-P6 donor cells truly have the highest rates of integration (rather than the highest rates of material transfer). Most lineage traced photoreceptors were rods, rather than cones, which is consistent with the timing of tamoxifen administration during the peak of rod genesis in the neonatal period. It would be interesting to expand our transcriptomic analysis to investigate the heterogeneity of embryonic timepoints, which contain early-born retinal precursor cells that may be better suited for cone regeneration. Importantly, we show that even at a single (P4) timepoint, photoreceptor precursor cells have a great degree of heterogeneity, suggesting that the identification of an optimal cell *identity*, rather than optimal chronologic age, may provide a more useful strategy for achieving transplantation success for the desired cell type.

Our unbiased scRNA-seq analysis revealed three transcriptionally distinct subpopulations of *Crx* + photoreceptor precursor cells present within the neonate mouse retina. The early (*Dll1+*) group is predominantly marked by genes that promote neuronal differentiation (*Pax6* (Kohwi et al., 2005), *Ascl1* (Brzezinski et al., 2011), *Neurog2* (Kowalchuk et al., 2018), *Hes6* (Bae et al., 2000)), suggesting that these are immature precursor cells committing to neuronal cell fates. The intermediate (*Neurod4+*) group is marked by pro-neural and visual function related genes (*Neurod4*, *Dll3*, *Abca4*). Notably, this group is also marked by the enrichment of genes associated with cytoskeletal dynamics and axon formation and guidance (*Tubb3*, *Tubb2b*, *Robo2*, *Gap43*), which suggests that these cells may be intrinsically poised for cell motility and migration–important prerequisites for transplantation success. Finally, the late (*Prom1+*) group is marked by genes related to photoreceptor maturation (*Nr2e3*, *Nrl*, *Prdm1*, *Rom1*) as well as synapse dynamics (*Dnm3*, *Snap25*), suggesting that they are committed photoreceptor precursors in the late-stages of differentiation. Interestingly, the early population acquired gene expression signatures of the intermediate and late groups over time, suggesting that these populations represent a spectrum of development with an increasing degree of photoreceptor maturation. These trends were further supported by our flow cytometry analysis, in which bulk-sorted tdTomato + samples showed sequential increases in the proportions of Prom1+/CD73+ cells over time. While cells from all three subpopulations contribute to the pool of mature photoreceptors, other retinal cell types were also labeled. To avoid inherent limitations of lineage tracing studies which rely on the fidelity of a single reporter (which in this study were enriched but not exclusive to the photoreceptor lineage) and that can reflect differences in promoter strength rather than true cellular abundance, it would be interesting in future studies to investigate only *Dll1*+, *Neurod4*+, and *Prom1*+ cells that co-express canonical photoreceptor markers, and to utilize single-cell resolution studies to definitively prove sequential maturation of these transition states.

Retinal organoids are an attractive source of photoreceptor precursor cells for retinal transplantation in human patients. Organoids recapitulate retinal development, can generate all retinal cell types, have scalable production, and can be generated from patient-derived cells to minimize immunogenicity. However, transplantation studies of cells from human retinal organoids exhibit similar limitations observed in mouse studies including limited donor cell integration and functional recovery. Furthermore, while many developmental patterns are shared between human and mouse, cross-species comparisons are critical to identify conserved pathways with therapeutic potential (Lu et al., 2020). By analyzing previously published human scRNA-seq datasets (Sridhar et al., 2020; Dorgau et al., 2024), we show that maturing human retinal organoids also have extensive heterogeneity of CRX + developing photoreceptor precursor cells that bear similarities to the *Crx +* subpopulations in the mouse. Our transcriptomic analysis suggests that these populations may be present as early as day (D) 45/60. Although organoids do not fully recapitulate *in vivo* retinal development, validating these findings in primary retinal organoids could reveal an earlier source of donor cells than the D200 or older retinal organoids commonly used for transplantation. Further investigation into the heterogeneity and dynamics of photoreceptor precursor cells in maturing human retinal organoids may help identify cell subpopulations with a greater capacity for integration.

Due to the overlapping nature of retinal development and the co-existence of the early, intermediate, and late photoreceptor cell populations at the same chronologic age, current donor cell isolation methods using broad photoreceptor markers cannot distinguish between these three groups. Ultimately, transplantation experiments using donor cells from isolated photoreceptor precursor populations will determine which has the highest rates of survival, synaptic connectivity, and functional integration within the retina. Based on our transcriptomic analysis, we hypothesize that the early (*Dll1+*) group may be ideally suited for neurogenesis and axon guidance, but these cells may require niche signaling and endogenous developmental cues that may be absent in diseased or degenerating retinas. The intermediate (*Neurod4+*) group in many ways represents an ideal donor cell population as it is marked by genes related to both photoreceptor development and neuronal cell migration and maturation. The late (*Prom1+*) group is most biased towards photoreceptor fate, but may have similar limitations as mature photoreceptors including poor cell survival following the mechanical stress of retinal dissociation and transplantation. The differentially expressed genes identified in our scRNA-seq data may also be leveraged to identify candidate genes to “prime” photoreceptor precursor cells prior to transplantation, or to direct the differentiation of human iPSCs or organoids towards an optimal cell type for neuronal integration. Further investigation into the genetic elements that regulate and drive the developmental transitions between states will improve our understanding of photoreceptor precursor maturation and may better inform future therapeutic strategies.

## Data Availability

Single-cell RNA-seq data have been deposited at the National Center for Biotechnology Information (NCBI) Gene Expression Omnibus (GEO) at GEO: accession number GSE297036, available at https://www.ncbi.nlm.nih.gov/geo/query/acc.cgi?acc=GSE297036.
