# Supplementary material for: Lineage tracing reveals photoreceptor precursor cell subpopulations that contribute to murine retinogenesis
Source: Front Cell Dev Biol. 2026 Jun 4;14:1814134. doi: 10.3389/fcell.2026.1814134 (PMC13276796; doi:10.3389/fcell.2026.1814134)
Supplement: Supplementary file 1 [file DataSheet7.pdf]

A

Dorgau 2024	
Age	# Cells
D10	7447
D20	5877
D33	10741
D45	13069
D60	11253
D90	5610
D150	7198
D210	3579

Sridhar 2020	
Age	# Cells
D45	2205
D60	4289
D90	576
D104	801
D110	1937
D205	5960

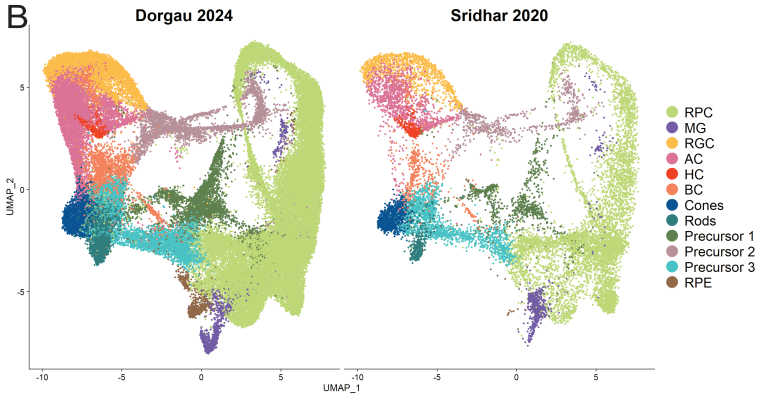

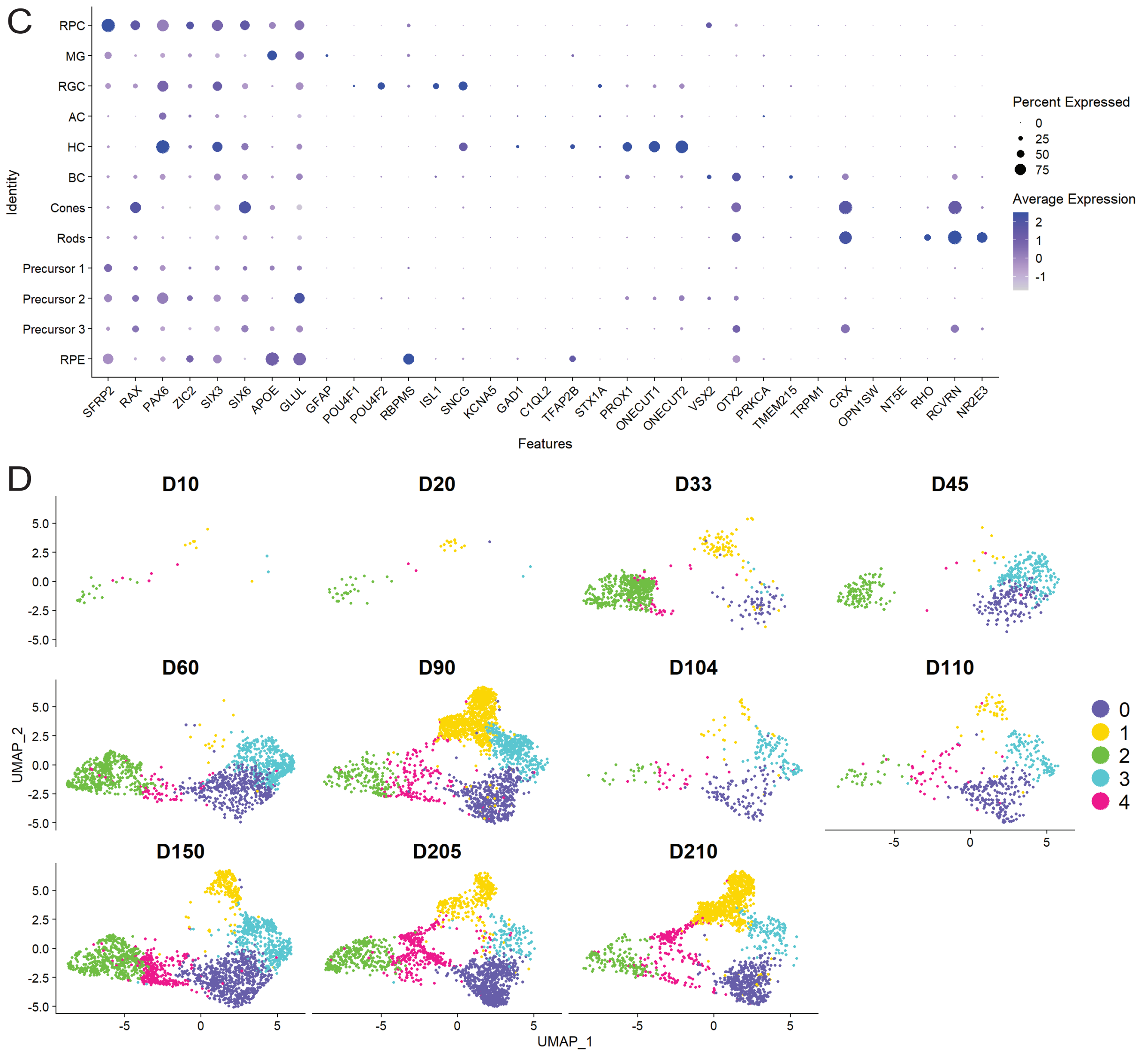

Figure S7. Generating a scRNA-seq dataset of maturing human retinal organoids.
A) Timepoints with number of cells used from published human retinal organoid scRNA-seq data (Dorgau et al., 2024, Sridhar et al., 2020). **B)** UMAP of integrated human retinal organoid scRNA-seq data faceted by data source. **C)** Dot plot of differentially expressed genes used to assign cluster identities. **D)** UMAP of human retinal organoid CRX precursor subset data faceted by sample timepoints. RPC, retinal progenitor cells; MG, Müller glia; RGC, retinal ganglion cells; AC,amacrine cells; HC, horizontal cells; BC, bipolar cells; RPE, retinal pigment epithelia.
