# Supplementary material for: Lineage tracing reveals photoreceptor precursor cell subpopulations that contribute to murine retinogenesis
Source: Front Cell Dev Biol. 2026 Jun 4;14:1814134. doi: 10.3389/fcell.2026.1814134 (PMC13276796; doi:10.3389/fcell.2026.1814134)
Supplement: Supplementary file 2 [file DataSheet2.pdf]

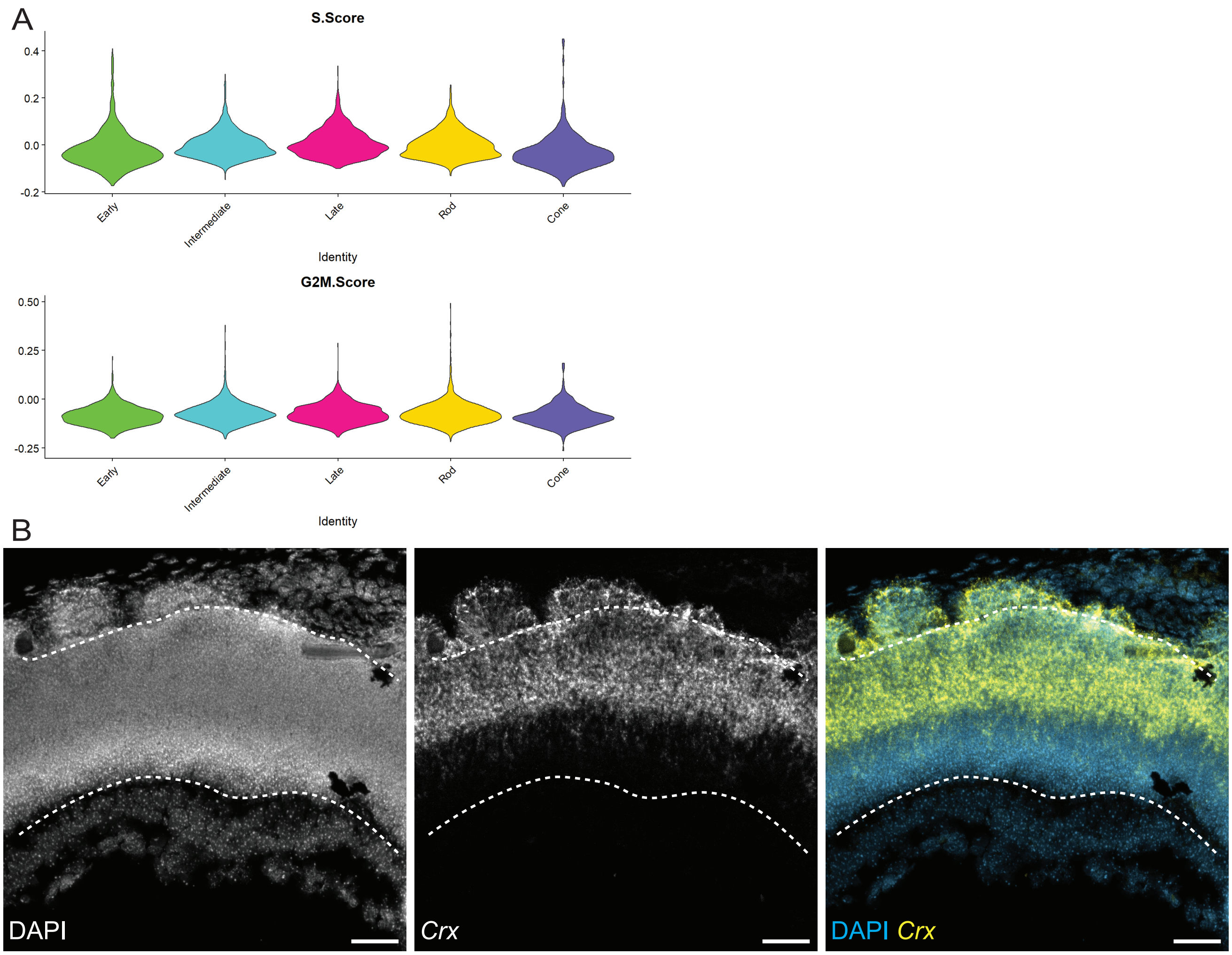

Figure S2. Characterization of neonatal Crx+ subpopulations.
A) Violin plots of S and G2M scores from cell cycle regression analysis of P2-P6 Crx+ subset populations. **B)** RNA-FISH analysis reveals spatial expression of Crx (yellow) in the P4 retina. DAPI nuclei counterstain used to define the boundaries of the neuroblast layer (dashed lines). Outer retina is oriented towards the top of the image. Scale bars = 50 μm.
