# Supplementary material for: Lineage tracing reveals photoreceptor precursor cell subpopulations that contribute to murine retinogenesis
Source: Front Cell Dev Biol. 2026 Jun 4;14:1814134. doi: 10.3389/fcell.2026.1814134 (PMC13276796; doi:10.3389/fcell.2026.1814134)
Supplement: Supplementary file 3 [file DataSheet4.pdf]

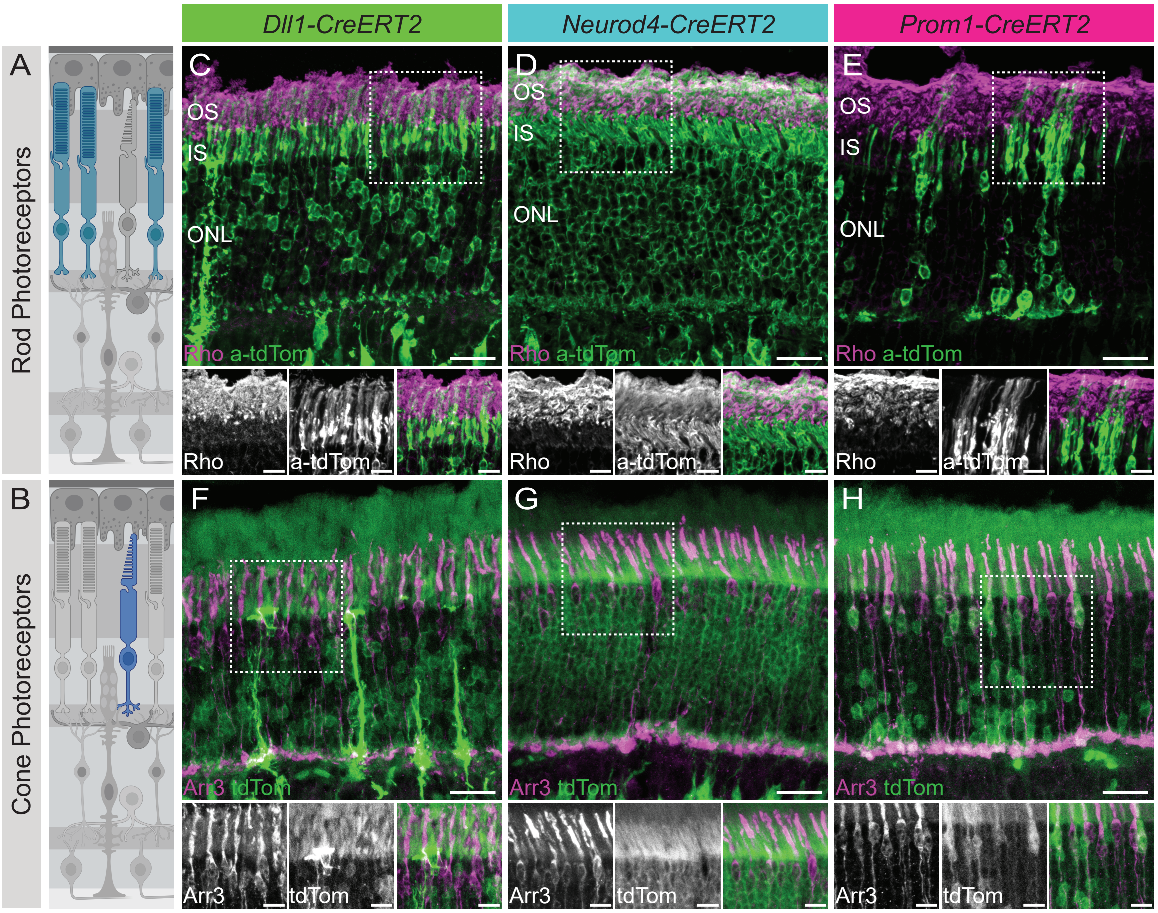

Figure S4. Labeled photoreceptor cells are primarily rods.
A-B) Diagram highlighting rod and cone photoreceptor cell location and morphology. Representative P30 retinas show co-expression of tdTomato+ lineage traced cells (green) with **C-E)** rhodopsin (Rho, magenta) or **F-H)** cone arrestin (Arr3, magenta). Scale bars = 20 μm. Dashed boxes outline magnified insets. Inset scale bars = 10 μm. ONL, outer nuclear layer; IS, inner segment; OS, outer segment.
