# Supplementary material for: Lineage tracing reveals photoreceptor precursor cell subpopulations that contribute to murine retinogenesis
Source: Front Cell Dev Biol. 2026 Jun 4;14:1814134. doi: 10.3389/fcell.2026.1814134 (PMC13276796; doi:10.3389/fcell.2026.1814134)
Supplement: Supplementary file 4 [file DataSheet6.pdf]

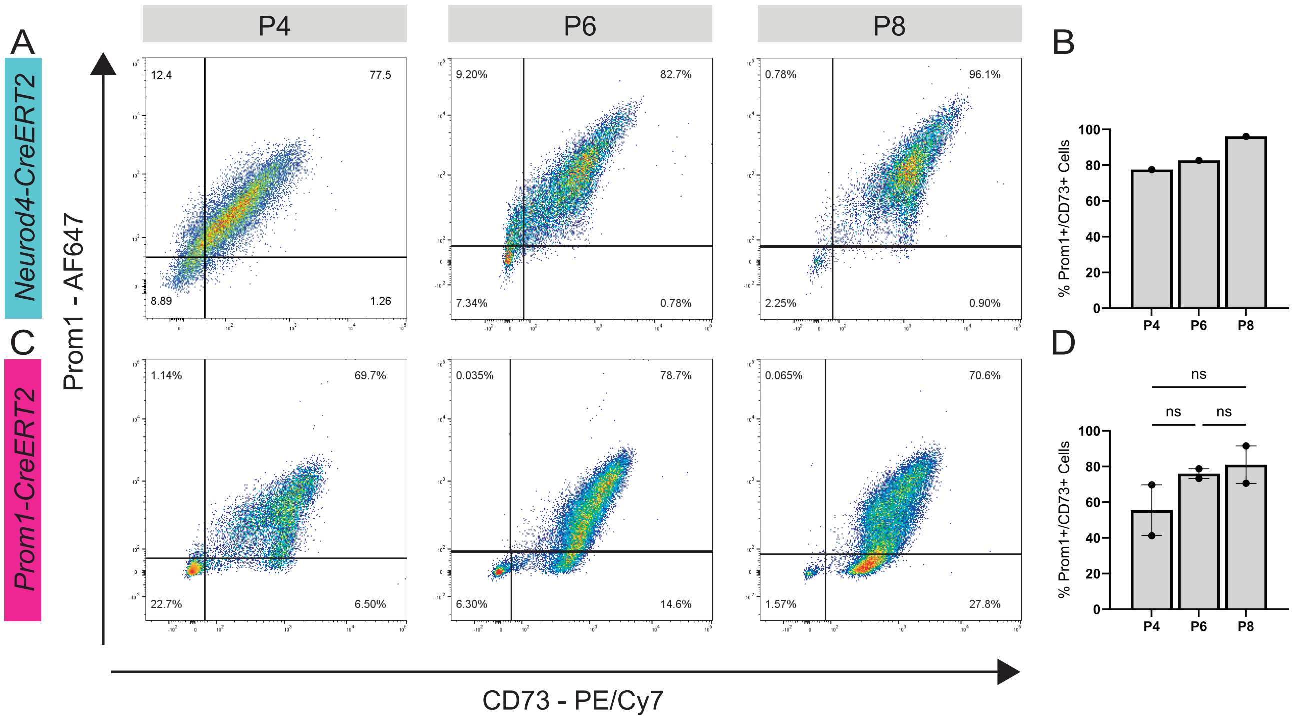

E

Prom1+/CD73+ Cells

Age	*Dll1-CreERT2*	*Neurod4-CreERT2*	*Prom1-CreERT2*
P4	44.0% ± 14.2%	77.5%	55.5% ± 14.3%
P6	77.3% ± 7.5%	82.7%	76.0% ± 2.7%
P8	89.4% ± 2.5%	96.1%	81.1% ± 10.5%

F

TdTomato+ Cells

Age	*Dll1-CreERT2*	*Neurod4-CreERT2*	*Prom1-CreERT2*
P4	17.5% ± 5.5%	30.6%	6.6% ± 1.3%
P6	15.7% ± 5.3%	57.2%	5.6% ± 2.6%
P8	14.8% ± 4.1%	62.3%	8.7% ± 2.2%

Figure S6. Flow cytometry analysis of Prom1 and CD73 expression in TdTomato+ labeled cells.
A-D) Dot plots of flow cytometry data of CD73-PE/Cy7 (x-axis) and Prom1-AF647 (y-axis) expression with quantification in Neurod4-CreERT2 **(A-B)**, and Prom1-CreERT2 **(C-D)** samples at P4, P6 and P8. Data were recorded from 10,000 tdTomato+ events. Quadrant gates were set up based on FMO control samples. Table summary of proportions of **E)** Prom1+/CD73+ cells and **F)** TdTomato+ cells in each lineage traced population at P4, P6, and P8. Values are means ± SEM (Dll1 n = 3, Neurod4 n = 1, Prom1 n =2).
