# Supplementary material for: Lineage tracing reveals photoreceptor precursor cell subpopulations that contribute to murine retinogenesis
Source: Front Cell Dev Biol. 2026 Jun 4;14:1814134. doi: 10.3389/fcell.2026.1814134 (PMC13276796; doi:10.3389/fcell.2026.1814134)
Supplement: Supplementary file 5 [file Table1.docx]

**Supplemental Table S1. Primer sequences used for PCR genotyping.**

| Primer Name | Sequence (5' -> 3') |
| --- | --- |
| Dll1-CreERT2-F | CCGGGCTGCCACGACCAA |
| Dll1-CreERT2-R | GGCGCGGCAACACCATTTTT |
| Neurod4-CreERT2-F | TAAAGATATCTCACGTACTGACGGTG |
| Neurod4-CreERT2-R | TCTCTGACCAGAGTCATCCTTAGC |
| Prom1-CreERT2-F | CAGGCTGTTAGCTTGGGTTC |
| Prom1-CreERT2-WT-R | TGCTGATTGCCTTCTGTCTG |
| Prom1-CreERT2-Mut-R | AGGCAAATTTTGGTGTACGG |
| Ai9-WT-F | AAGGGAGCTGCAGTGGAGTA |
| Ai9-WT-R | CCGAAAATCTGTGGGAAGTC |
| Ai9-Mut-F | CTGTTCCTGTACGGCATGG |
| Ai9-Mut-R | GGCATTAAAGCAGCGTATCC |
