# Supplementary material for: Lineage tracing reveals photoreceptor precursor cell subpopulations that contribute to murine retinogenesis
Source: Front Cell Dev Biol. 2026 Jun 4;14:1814134. doi: 10.3389/fcell.2026.1814134 (PMC13276796; doi:10.3389/fcell.2026.1814134)
Supplement: Supplementary file 6 [file DataSheet3.pdf]

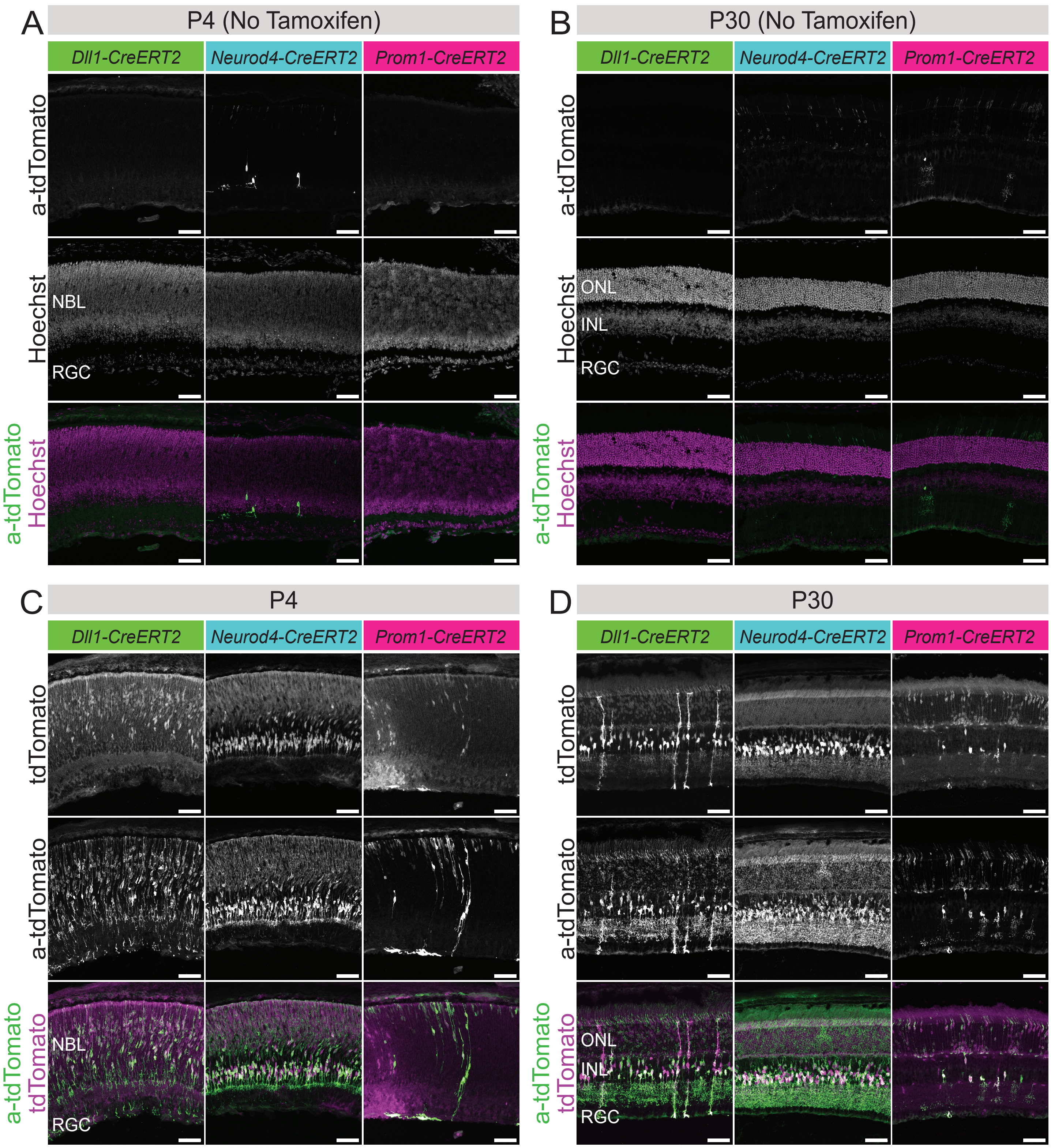

Figure S3. Untreated lineage tracing samples have minimal background labeling.
A-B) Representative immunofluorescence images of P4 and P30 retinas from control samples that were not treated with tamoxifen show rare tdTomato+ cells (green = anti-tdTomato; magenta = Hoechst). Scale bars = 50 μm. **C-D)** Representative immunofluorescence images of P4 and P30 retinas showing co-expression of endogenous tdTomato and anti-RFP antibody signal (green = anti-tdTomato; magenta = endogenous tdTomato). Scale bars = 50 μm.
