# Supplementary material for: Lineage tracing reveals photoreceptor precursor cell subpopulations that contribute to murine retinogenesis
Source: Front Cell Dev Biol. 2026 Jun 4;14:1814134. doi: 10.3389/fcell.2026.1814134 (PMC13276796; doi:10.3389/fcell.2026.1814134)
Supplement: Supplementary file 7 [file Table2.docx]

**Supplemental Table S2. Primer sequences used for qPCR analysis.**

| Gene | Forward Primer (5’ -> 3’) | Reverse Primer (5’ -> 3’) |
| --- | --- | --- |
| *Abca4* | CCCCTGGAAGGGGATGAGTT | CTCTGGTCGAACAACCTTCAC |
| *Asc1* | CCACCATCTCCCCCAACTAC | ATCTGCTGCCATCCTGCTT |
| *B2m* | CTGTATGCTATCCAGAAAACCCC | AGTGGGGGTGAATTCAGTGT |
| *Cadm3* | GCCAAGTCCCTTGTCACTGT | ATTCGTGTTTGGTCCCCGTG |
| *Ccdc136* | CCAAGCAGAAGGAGCTCACA | TCCCAAACATGTTCTCGCACT |
| *Ccnd1* | CCACAGATGTGAAGTTCATTTCCAA | GGGGGTCCTTGTTTAGCCAG |
| *Cd24a* | GCAGATCTCCACTTACCGAACA | GGTTGCAGTAAATCTGCGTGG |
| *Cmtm7* | CAAAGTGGCGCAGATGGTCA | CAAAGATCGACAGGGGCCAG |
| *Cnp* | AAGCTGGCCAGGTCTTTCTG | TCTCTTCACCACCTCCTGCT |
| *Crx* | GCCTCACTATTCAGTCAATGCC | GCTCCTGGTGAATGTGGTC |
| *Crym* | GTGCTGTATGTGGACTCCCG | CTTCCACTGCCATCCCCAAA |
| *Dkk3* | ACCAACAACCAGAGTGGACA | TGTCTCGGGTGCATAGCATCT |
| *Dll1* | CCAGTACTGCACTGACCCAA | ATTCCTGCACGGCTTATGGT |
| *Dll3* | GGGCAGCTGTAGTGAAACCT | CTTCACCGCCAACACACAAG |
| *Dnm3* | GGTCTACCCAGATAAATCTGTAGGG | CCACTTGCCTCTCCAACTGT |
| *Fabp7* | GGTTCGGTTGGATGGAGACA | AGAGTCACGACCATCTTGCC |
| *Gngt2* | ACCCACGTGATCTGATTTCCAA | AGCTTAGCACACAAGTGCCT |
| *Hes5* | GGAGAAAAACCGACTGCGGA | TAGTCCTGGTGCAGGCTCTT |
| *Igfbp2* | GGTGCCAAACACCTCAGTCT | TTCAGAGACATCTTGCACTGCT |
| *Neurod4* | TGGAACTGGACTTCCAGAGAATC | AGAGCCCGGTCTTCTCTCTT |
| *Neurog2* | CCGCGTAGGATGTTCGTCA | CTGGAGGACATCGGGGTCAG |
| *Nrl* | GCACGGGGAAAAGGGACGAC | TGATGATGTAATGGCAGAGATGACG |
| *Nt5e* | GAAACCTGATCTGTGATGCCA | TGCCACCTCCGTTTACAATG |
| *Opn1sw* | TGTCTCTGCTACGTGCCCTA | AGCCCGGAACTGCTTATTCAT |
| *Pias3* | AGCGTGAGGTGGACATGC | GGCAGAACCTCCCTGGAC |
| *Pon2* | ACCTCATTAAAGGAATCGAAACTGG | ATCAAAGCCCCAGCTGACTC |
| *Prom1* | AGAATTCGCTCAGCAGCAGT | CATCAATTGTCGTATACCCCCTT |
| *Prph2* | TCGGCGCACTACAGCTATG | GCAGTGATGCTCACCTCAAAG |
| *Rgs16* | CAACACCTGCCTGGAGAGAG | AGGCAGCCACCCCATTTTTA |
| *Rho* | TTGGCTGGTCCAGGTACATC | TAACCATGCGGGTGACTTCC |
| *Rlbp1* | GGATTCATTCCCAGCCAGGT | TCCGTGAACAAAGACCCTCTG |
| *Robo2* | CAGCTCAGAAAGGAGTGGCA | AGCTGTCACTGTCATAGCCATT |
| *Rom1* | CAGGCCAAACGTCTGATGGA | GCTCTGGATCCGGTCAACTAC |
| *Sag* | TCTCGGGACAAGTCGGTGAC | AAGGTCAGGCCCATCACATC |
| *Snap25* | TGGATGAGCAAGGCGAACAA | TATGGCGGAGGTTTCCGATG |
| *Trib2* | TCGCATTGCGTTTCTTGCAT | CAGCTGATCTCAAACACCTTGC |
| *Tspan15* | TGCATCAGGAACACGACAGA | AACTGAGGAAGCAGGATGCC |
| *Tubb2b* | CCAGATCGGTGCCAAGTTTT | CCTGACTGAGTCCATTGTGC |
| *Tubb3* | GCCAAGTTCTGGGAGGTCAT | GTCGGGCCTGAATAGGTGTC |
| *Vxn* | CCTTCCAAGGTGTCCAGTTCA | GTTGAGGCAGTAGCTCCAGG |
