# Supplementary material for: Lineage tracing reveals photoreceptor precursor cell subpopulations that contribute to murine retinogenesis
Source: Front Cell Dev Biol. 2026 Jun 4;14:1814134. doi: 10.3389/fcell.2026.1814134 (PMC13276796; doi:10.3389/fcell.2026.1814134)
Supplement: Supplementary file 8 [file DataSheet1.pdf]

A

Age	No. Sequenced Cells
E14	15922
E16	2973
P0	10590
P3.1	12702
P3.2	3239
P6	13856

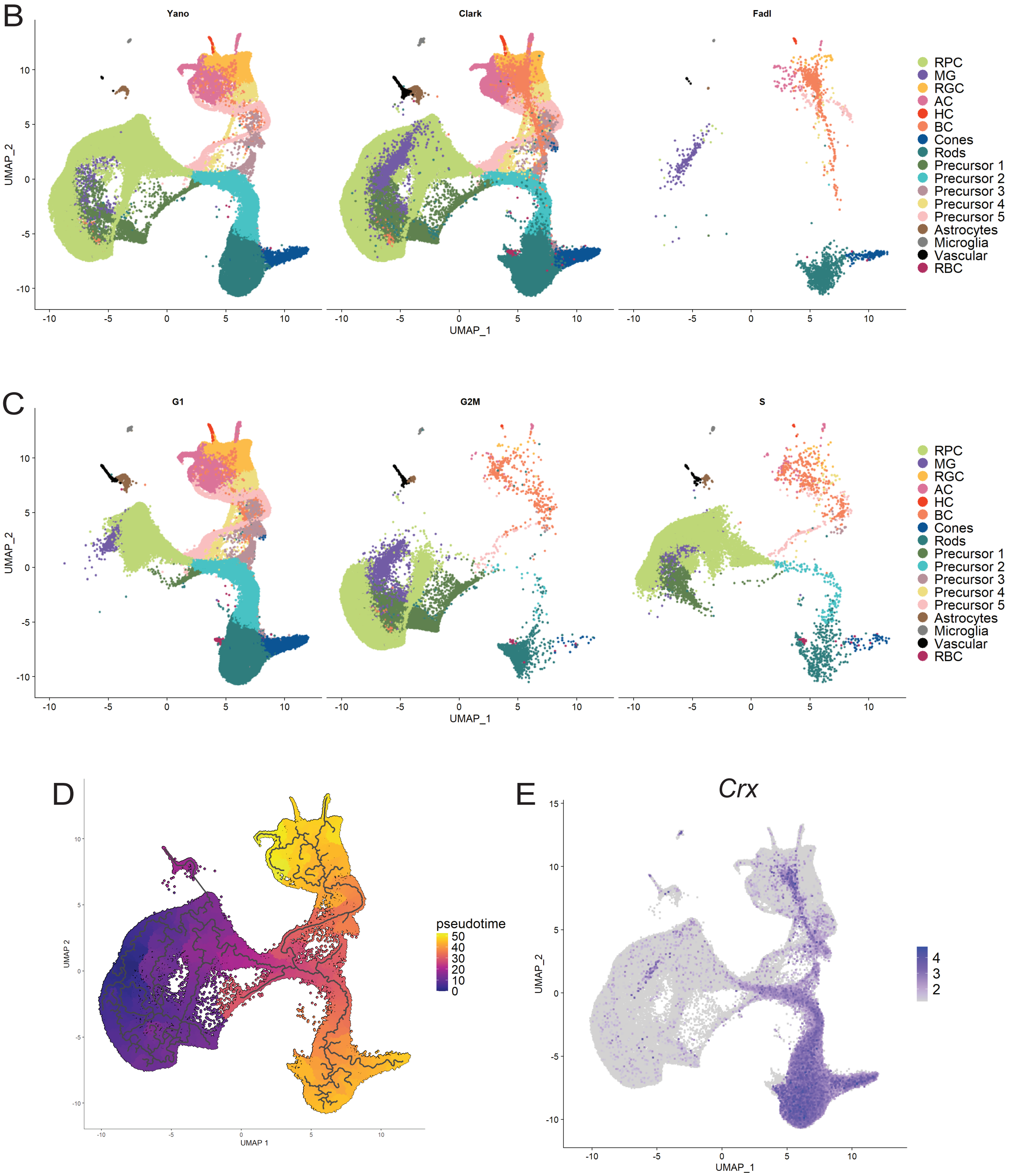

Figure S1. Integrating retinal scRNA-seq data to generate time-resolved atlas of mouse retinal development. A) Ages and number of cells sequenced for primary scRNA-seq data generated in this study. **B-C)** UMAP of integrated scRNA-seq data faceted by data source **(B)** and cell cycle regression analysis **(C)**. **D)** Pseudotime trajectory analysis of mouse retina dataset using Monocle3, using Ccnd1 as a reference. **E)** UMAP of Crx expression in the E11-P60 mouse retinal development data.
