# Supplementary material for: Lineage tracing reveals photoreceptor precursor cell subpopulations that contribute to murine retinogenesis
Source: Front Cell Dev Biol. 2026 Jun 4;14:1814134. doi: 10.3389/fcell.2026.1814134 (PMC13276796; doi:10.3389/fcell.2026.1814134)
Supplement: Supplementary file 9 [file Table5.docx]

**Supplemental Table S5. Top 100 differentially expressed genes from Late cluster.**

| geneID | avg_log2FC | p_val_adj |
| --- | --- | --- |
| *Gngt2* | 1.464645 | 0 |
| *Tmem158* | 1.235338 | 1.45E-116 |
| *Ece1* | 1.218809 | 1.45E-121 |
| *Ccdc136* | 1.181693 | 4.17E-110 |
| *Lcorl* | 1.131553 | 8.44E-106 |
| *Rtl8a* | 1.121339 | 1.53E-68 |
| *Insm2* | 1.076083 | 9.26E-84 |
| *Hmgn2* | 1.069173 | 2.78E-139 |
| *Chgb* | 1.060216 | 4.70E-150 |
| *Slc25a33* | 1.033072 | 1.16E-98 |
| *Ndfip1* | 1.013956 | 3.55E-105 |
| *Pcp2* | 1.004091 | 2.48E-81 |
| *Dnm3* | 0.998308 | 6.04E-57 |
| *Dmd* | 0.996735 | 2.23E-120 |
| *Nrl* | 0.979795 | 7.70E-163 |
| *Agpat3* | 0.955483 | 7.04E-119 |
| *Dusp8* | 0.920273 | 1.08E-46 |
| *Glo1* | 0.919568 | 1.51E-165 |
| *Fabp5* | 0.904833 | 2.92E-38 |
| *Amer2* | 0.85237 | 1.97E-109 |
| *Clstn1* | 0.841183 | 3.81E-77 |
| *C2cd4b* | 0.833723 | 2.96E-49 |
| *Meis2* | 0.827876 | 1.15E-82 |
| *Dusp26* | 0.794894 | 4.80E-44 |
| *C1qtnf12* | 0.79219 | 2.11E-51 |
| *Tma7* | 0.789737 | 4.21E-135 |
| *Tmem86a* | 0.786479 | 5.94E-50 |
| *Nxph4* | 0.768328 | 6.54E-43 |
| *Ccdc175* | 0.765297 | 5.12E-50 |
| *Ppfia2* | 0.762093 | 1.50E-37 |
| *Cadm2* | 0.751603 | 2.61E-65 |
| *Podxl2* | 0.75059 | 1.88E-72 |
| *Arhgap31* | 0.748741 | 1.34E-71 |
| *Inhbb* | 0.74711 | 1.31E-46 |
| *Hcn1* | 0.741104 | 7.20E-44 |
| *Nfic* | 0.737136 | 3.11E-41 |
| *Apoe* | 0.736961 | 2.43E-25 |
| *Cxxc5* | 0.731054 | 3.30E-57 |
| *Baiap2* | 0.727135 | 2.30E-45 |
| *Lgmn* | 0.722413 | 2.32E-41 |
| *Pgm1* | 0.716946 | 1.76E-38 |
| *Rasl11a* | 0.716728 | 2.52E-43 |
| *Zc3h14* | 0.710525 | 5.22E-43 |
| *Kcne1l* | 0.709848 | 1.26E-30 |
| *Gnb3* | 0.698809 | 1.95E-91 |
| *Cltb* | 0.696888 | 1.01E-69 |
| *Proser2* | 0.686124 | 4.77E-34 |
| *Atp6v0b* | 0.685913 | 1.08E-81 |
| *Gm17167* | 0.685255 | 1.31E-29 |
| *Htra1* | 0.682167 | 6.31E-35 |
| *Cplx3* | 0.680701 | 4.85E-47 |
| *Cplx2* | 0.679846 | 1.95E-59 |
| *Malat1* | 0.678956 | 7.38E-229 |
| *Plxna2* | 0.67498 | 6.19E-23 |
| *Grina* | 0.674609 | 4.58E-50 |
| *Hist1h1c* | 0.671498 | 1.90E-21 |
| *Fhod3* | 0.671033 | 4.66E-31 |
| *Prdm1* | 0.669605 | 2.64E-32 |
| *Rbp3* | 0.668803 | 4.69E-63 |
| *Pik3r1* | 0.662095 | 8.62E-78 |
| *Kpna3* | 0.661252 | 2.33E-22 |
| *Epha8* | 0.660755 | 6.14E-27 |
| *Tmem176a* | 0.660728 | 5.25E-28 |
| *Actg1* | 0.656466 | 2.72E-43 |
| *Nr2e3* | 0.655586 | 1.13E-69 |
| *Jund* | 0.649451 | 9.37E-53 |
| *Snap25* | 0.639638 | 7.94E-53 |
| *Trak2* | 0.637677 | 3.94E-25 |
| *Ldha* | 0.636883 | 4.58E-111 |
| *Stmn1* | 0.636281 | 6.68E-52 |
| *Cited2* | 0.633746 | 2.54E-29 |
| *Smim13* | 0.629231 | 3.03E-24 |
| *Unc119* | 0.627355 | 4.96E-96 |
| *Apbb1* | 0.627006 | 1.69E-42 |
| *Atp6v0c* | 0.626086 | 6.17E-33 |
| *Lmo1* | 0.617194 | 5.52E-20 |
| *Acsl1* | 0.611369 | 4.53E-25 |
| *Klf10* | 0.610091 | 6.38E-22 |
| *Dleu2* | 0.603914 | 2.27E-14 |
| *Bin1* | 0.602704 | 5.49E-27 |
| *Prcd* | 0.600512 | 2.08E-34 |
| *Tob2* | 0.59693 | 1.62E-20 |
| *Glcci1* | 0.585299 | 5.59E-32 |
| *Egflam* | 0.584399 | 7.11E-22 |
| *Gm4258* | 0.584176 | 8.09E-18 |
| *Cabp1* | 0.580831 | 1.03E-19 |
| *Samd11* | 0.57521 | 6.07E-52 |
| *Mxi1* | 0.573926 | 5.37E-17 |
| *Ptpn21* | 0.571918 | 1.33E-17 |
| *Hspa12a* | 0.570573 | 1.85E-24 |
| *Mtfp1* | 0.568002 | 3.49E-19 |
| *Oip5os1* | 0.566538 | 9.33E-18 |
| *Tecpr1* | 0.565926 | 4.99E-20 |
| *Rnasek* | 0.561867 | 3.05E-18 |
| *Myo9a* | 0.559454 | 1.29E-15 |
| *Rsrp1* | 0.557868 | 2.98E-39 |
| *Btbd9* | 0.556373 | 4.74E-15 |
| *Rom1* | 0.553804 | 4.80E-103 |
| *Gse1* | 0.552554 | 2.67E-15 |
| *Mak* | 0.551017 | 8.00E-21 |
