# Supplementary material for: Lineage tracing reveals photoreceptor precursor cell subpopulations that contribute to murine retinogenesis
Source: Front Cell Dev Biol. 2026 Jun 4;14:1814134. doi: 10.3389/fcell.2026.1814134 (PMC13276796; doi:10.3389/fcell.2026.1814134)
Supplement: Supplementary file 10 [file DataSheet5.pdf]

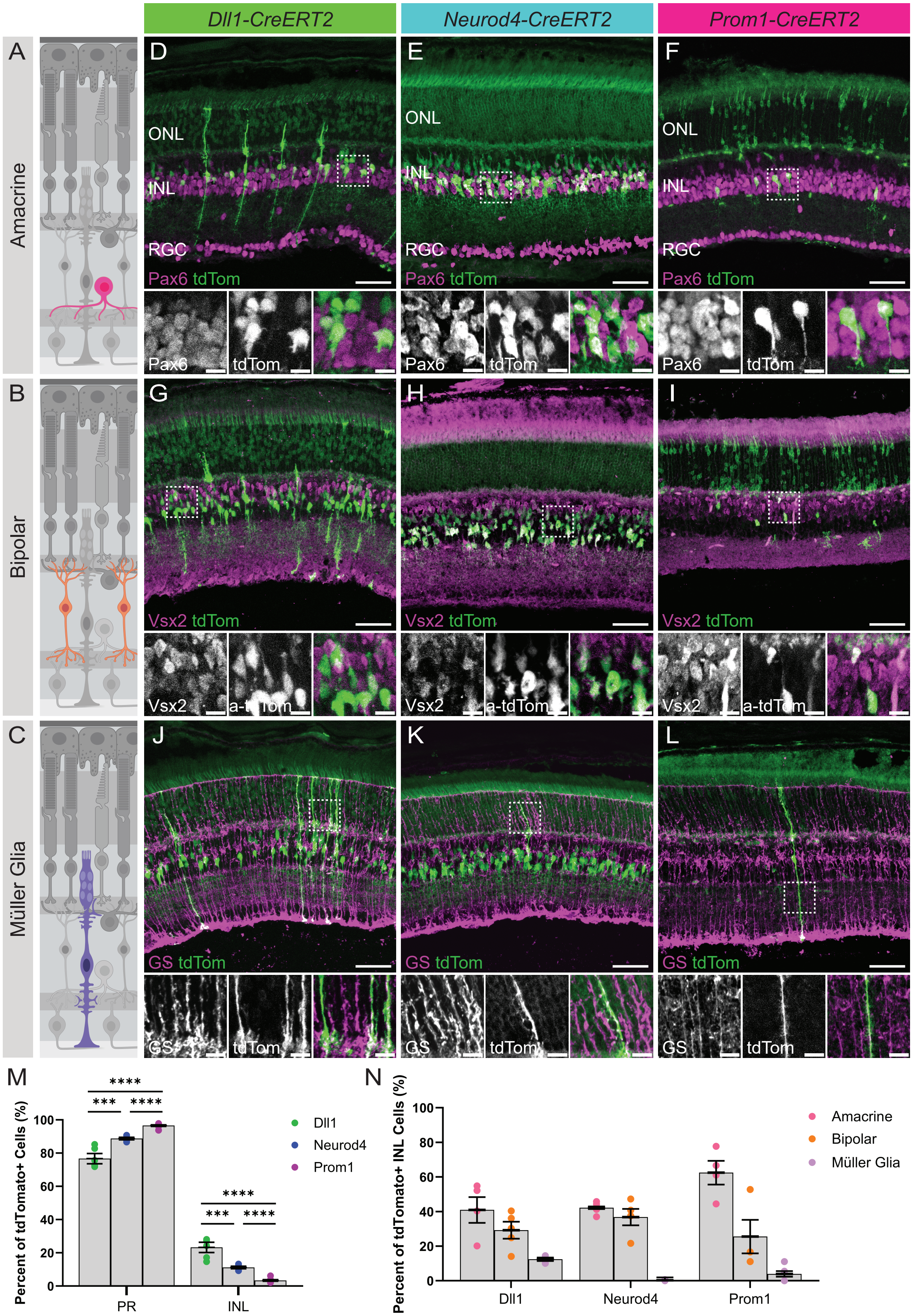

Figure S5. Immunofluorescence analysis identifies labeled INL cells. A-C) Diagrams highlighting amacrine, bipolar, and Müller glia cell location and morphology. Representative P30 retinas show co-staining of tdTomato+ lineage traced cells (green) with **D-F)** Pax6 (amacrine cell marker, magenta), **G-I)** Vsx2 (bipolar cell marker, magenta), or **J-L)** Glutamine Synthetase (GS, Müller glia marker, magenta). Scale bars = 50 μm. Dashed boxes outline magnified insets. Inset scale bars = 10 μm. ONL, outer nuclear layer; INL, inner nuclear layer; RGC, retinal ganglion cell. **M)** Percentage of tdTomato+ cells in the ONL (photoreceptors, PR) vs. INL at P30, relative to the total number of tdTomato+ cells. Dll1 76.7% ONL / 23.3% INL; Neurod4 88.8% ONL / 11.2% INL; Prom1 96.6% ONL / 3.4% INL (n=5-6 eyes per condition). Data represented as mean ± SEM. ***p=0.0001, ****p<0.0001. **N)** Percentage of tdTomato+ INL cells co-expressing amacrine (pink), bipolar (orange), or Müller glia (purple) cell markers in each lineage traced subpopulation. Dll1 40.9% amacrine, 29.3% bipolar, and 12.4% Müller glia cells; Neurod4 42.2% amacrine, 36.8% bipolar, and 0.3% Müller glia cells; Prom1 62.5% amacrine, 25.5% bipolar, and 4.0% Müller glia cells (n=3-5 eyes per condition). Data represented as mean ± SEM.
