# Supplementary material for: Lineage tracing reveals photoreceptor precursor cell subpopulations that contribute to murine retinogenesis
Source: Front Cell Dev Biol. 2026 Jun 4;14:1814134. doi: 10.3389/fcell.2026.1814134 (PMC13276796; doi:10.3389/fcell.2026.1814134)
Supplement: Supplementary file 11 [file Table3.docx]

**Supplemental Table S3. Top 100 differentially expressed genes from Early cluster.**

| geneID | avg_log2FC | p_val_adj |
| --- | --- | --- |
| *Hes5* | 3.252023 | 2.64E-242 |
| *Ccnd1* | 2.946624 | 0 |
| *Dbi* | 2.774712 | 0 |
| *Crym* | 2.701497 | 0 |
| *Ptn* | 2.587425 | 0 |
| *Miat* | 2.290623 | 0 |
| *Hes1* | 2.267712 | 3.86E-159 |
| *Neurog2* | 2.238715 | 0 |
| *Gadd45g* | 2.188985 | 1.34E-299 |
| *Ascl1* | 1.928221 | 0 |
| *Mdk* | 1.815635 | 8.12E-297 |
| *Espn* | 1.812779 | 0 |
| *Pfn2* | 1.81073 | 7.94E-180 |
| *Dapl1* | 1.803178 | 2.07E-209 |
| *Hes6* | 1.801488 | 0 |
| *Id3* | 1.772161 | 2.76E-109 |
| *Rgs16* | 1.769421 | 2.80E-239 |
| *Dkk3* | 1.758085 | 6.97E-251 |
| *Id1* | 1.745463 | 9.21E-116 |
| *Rbp1* | 1.693638 | 2.79E-274 |
| *E130114P18Rik* | 1.680931 | 7.36E-281 |
| *Kdr* | 1.671604 | 1.31E-286 |
| *Ier2* | 1.663061 | 2.96E-135 |
| *Pax6* | 1.631502 | 5.95E-228 |
| *Sat1* | 1.62759 | 2.01E-80 |
| *Fos* | 1.613072 | 1.99E-86 |
| *Tcf4* | 1.580316 | 2.24E-241 |
| *Sox11* | 1.576485 | 3.92E-205 |
| *E2f1* | 1.563082 | 4.45E-240 |
| *Klf13* | 1.542134 | 2.05E-178 |
| *Fbxw7* | 1.537211 | 1.45E-213 |
| *Prdx6* | 1.531805 | 5.77E-219 |
| *Csrp2* | 1.508376 | 4.78E-221 |
| *Dll1* | 1.505097 | 2.08E-225 |
| *Ncald* | 1.415248 | 4.13E-231 |
| *Egr1* | 1.402643 | 1.69E-134 |
| *Mfap2* | 1.375317 | 9.10E-196 |
| *Sparc* | 1.373114 | 8.01E-181 |
| *Zfp36l1* | 1.361516 | 2.19E-188 |
| *Cnp* | 1.350496 | 9.42E-160 |
| *Acsl3* | 1.340115 | 7.61E-109 |
| *Pon2* | 1.329392 | 1.89E-246 |
| *Myb* | 1.30723 | 4.55E-197 |
| *Gstm5* | 1.296367 | 1.20E-134 |
| *Tgfb2* | 1.263311 | 1.12E-161 |
| *Ass1* | 1.25786 | 5.79E-186 |
| *Ldhb* | 1.254749 | 1.77E-136 |
| *Prdx1* | 1.251233 | 1.57E-139 |
| *Tfdp2* | 1.247461 | 4.94E-126 |
| *Mybl1* | 1.219211 | 3.10E-113 |
| *Tbx3* | 1.21717 | 9.40E-169 |
| *Mt1* | 1.211418 | 4.41E-82 |
| *Ppp1r14b* | 1.206789 | 1.60E-88 |
| *Cdk4* | 1.193782 | 5.20E-155 |
| *Glul* | 1.190984 | 1.73E-110 |
| *Sox4* | 1.177009 | 1.70E-192 |
| *Sorbs2* | 1.163742 | 7.12E-100 |
| *Rlbp1* | 1.157246 | 4.85E-150 |
| *Jam3* | 1.152075 | 1.20E-132 |
| *Dhx32* | 1.149092 | 2.29E-145 |
| *Dll3* | 1.14794 | 1.29E-112 |
| *Prss23* | 1.136455 | 1.43E-120 |
| *Tox3* | 1.132045 | 1.11E-102 |
| *Nme1* | 1.125417 | 1.77E-130 |
| *Nme2* | 1.121926 | 1.28E-81 |
| *Nnat* | 1.111991 | 8.99E-112 |
| *Tead2* | 1.111019 | 7.62E-128 |
| *Carhsp1* | 1.107567 | 9.25E-115 |
| *Crip2* | 1.097947 | 4.94E-113 |
| *Olig2* | 1.082153 | 3.75E-170 |
| *Gnai2* | 1.081701 | 7.62E-86 |
| *Cp* | 1.075351 | 4.80E-109 |
| *Gnb2l1* | 1.072009 | 1.34E-48 |
| *Prox1* | 1.068172 | 6.42E-137 |
| *Tspan15* | 1.067431 | 4.87E-95 |
| *Lrrfip1* | 1.065753 | 2.90E-147 |
| *Rbfox2* | 1.061682 | 7.50E-134 |
| *Cd24a* | 1.057107 | 5.57E-111 |
| *Rpsa* | 1.056557 | 2.83E-231 |
| *Rps3a1* | 1.054246 | 1.89E-251 |
| *Sh3bgrl* | 1.045534 | 7.40E-141 |
| *Cmtm7* | 1.044414 | 2.38E-106 |
| *Cd9* | 1.04022 | 4.18E-137 |
| *Rplp0* | 1.039119 | 2.58E-259 |
| *Irx5* | 1.038072 | 1.15E-142 |
| *Olfm1* | 1.030981 | 2.10E-89 |
| *Rpl36a* | 1.026741 | 1.33E-176 |
| *Dpysl4* | 1.024886 | 5.29E-92 |
| *Cotl1* | 1.01873 | 2.17E-78 |
| *Mfge8* | 1.017953 | 8.24E-123 |
| *Rps18* | 1.008245 | 5.49E-247 |
| *Rcn2* | 1.007272 | 5.72E-77 |
| *Npc2* | 1.004248 | 8.65E-105 |
| *Gas1* | 0.996824 | 1.59E-77 |
| *Rrs1* | 0.996382 | 3.73E-77 |
| *Tox* | 0.994667 | 9.87E-90 |
| *Gpm6a* | 0.992092 | 1.09E-73 |
| *2610001J05Rik* | 0.983402 | 3.96E-65 |
| *Id2* | 0.978781 | 7.63E-54 |
| *Glrx3* | 0.978386 | 9.34E-93 |
