# Supplementary material for: Lineage tracing reveals photoreceptor precursor cell subpopulations that contribute to murine retinogenesis
Source: Front Cell Dev Biol. 2026 Jun 4;14:1814134. doi: 10.3389/fcell.2026.1814134 (PMC13276796; doi:10.3389/fcell.2026.1814134)
Supplement: Supplementary file 12 [file Table4.docx]

**Supplemental Table S4. Top 100 differentially expressed genes from Intermediate cluster.**

| geneID | avg_log2FC | p_val_adj |
| --- | --- | --- |
| *Fabp7* | 2.132516 | 8.35E-181 |
| *Neurod4* | 1.927713 | 0 |
| *Tubb3* | 1.819868 | 9.16E-280 |
| *Tubb2b* | 1.364299 | 2.95E-194 |
| *Pak3* | 1.286075 | 2.30E-192 |
| *Tagln3* | 1.103163 | 1.48E-118 |
| *3110035E14Rik* | 1.103106 | 3.89E-60 |
| *Sox4* | 1.051607 | 3.45E-220 |
| *Robo2* | 1.038896 | 2.16E-93 |
| *Trib2* | 1.036128 | 4.88E-112 |
| *Nol4* | 1.013876 | 1.30E-120 |
| *Bnip3* | 1.000382 | 2.64E-70 |
| *Btbd17* | 0.998782 | 1.78E-122 |
| *Mgarp* | 0.961147 | 9.69E-78 |
| *Tuba1a* | 0.954757 | 4.84E-256 |
| *Gap43* | 0.9542 | 6.20E-102 |
| *Lhx4* | 0.951022 | 1.48E-92 |
| *Mybl1* | 0.945043 | 1.09E-72 |
| *Ppib* | 0.928486 | 4.08E-148 |
| *Igfbp2* | 0.919402 | 9.39E-41 |
| *Serpinh1* | 0.914781 | 7.72E-60 |
| *Mex3a* | 0.907133 | 6.39E-84 |
| *Ptgds* | 0.89789 | 3.39E-77 |
| *Stmn2* | 0.892867 | 1.05E-50 |
| *Tpi1* | 0.859665 | 1.23E-81 |
| *Cd24a* | 0.855412 | 2.56E-67 |
| *Zfos1* | 0.837872 | 1.53E-24 |
| *Scd2* | 0.836288 | 1.41E-80 |
| *Meg3* | 0.836156 | 3.29E-68 |
| *Abca4* | 0.826471 | 2.71E-66 |
| *Tmcc3* | 0.817729 | 3.75E-78 |
| *Micu1* | 0.81213 | 3.70E-68 |
| *Ndufa4l2* | 0.805778 | 4.03E-31 |
| *Ppp1r14b* | 0.798544 | 3.86E-59 |
| *Vxn* | 0.784175 | 1.96E-109 |
| *Tubb5* | 0.780425 | 1.12E-131 |
| *Eef2* | 0.763797 | 2.09E-87 |
| *Cnn3* | 0.761473 | 7.88E-68 |
| *Abcd2* | 0.753783 | 8.93E-81 |
| *Gem* | 0.75291 | 1.02E-61 |
| *Stmn1* | 0.75043 | 2.40E-15 |
| *Khdrbs3* | 0.747429 | 8.95E-49 |
| *Basp1* | 0.743911 | 3.75E-41 |
| *Dhx32* | 0.738489 | 5.30E-62 |
| *Fscn1* | 0.731692 | 4.72E-61 |
| *Soga3* | 0.724842 | 7.30E-50 |
| *Enho* | 0.721133 | 2.14E-50 |
| *Vim* | 0.706015 | 4.14E-48 |
| *Dll3* | 0.701894 | 5.69E-50 |
| *Cadm3* | 0.701348 | 8.11E-46 |
| *Marcksl1* | 0.693069 | 1.15E-68 |
| *Hn1* | 0.689576 | 3.26E-16 |
| *Hist3h2ba* | 0.688436 | 3.33E-55 |
| *Jpt1* | 0.684257 | 1.40E-26 |
| *Nlgn1* | 0.684213 | 1.47E-51 |
| *Cdk2ap1* | 0.680478 | 5.65E-40 |
| *Vsx2* | 0.677917 | 1 |
| *Fam162a* | 0.673905 | 3.86E-22 |
| *Ddah2* | 0.669966 | 1.99E-60 |
| *Glcci1* | 0.66882 | 6.02E-29 |
| *Tpd52* | 0.660618 | 1.23E-59 |
| *Rorb* | 0.647976 | 7.28E-108 |
| *Eef1a1* | 0.646117 | 2.76E-164 |
| *Pgk1* | 0.64601 | 4.56E-17 |
| *Clvs1* | 0.64119 | 1.28E-59 |
| *Rnd3* | 0.639091 | 1.57E-41 |
| *Zeb2* | 0.638408 | 1.47E-37 |
| *Dbn1* | 0.628669 | 1.10E-37 |
| *Trp53i11* | 0.625812 | 2.43E-38 |
| *Rps9* | 0.622979 | 3.27E-158 |
| *Npc2* | 0.620947 | 4.13E-39 |
| *Dusp5* | 0.616687 | 7.46E-30 |
| *Gadd45g* | 0.616275 | 1.50E-115 |
| *Smad2* | 0.613881 | 1.14E-37 |
| *Rasl10b* | 0.613513 | 1.52E-54 |
| *Hnrnpa1* | 0.613028 | 6.22E-24 |
| *Mir124-2hg* | 0.604644 | 1.06E-28 |
| *Scg5* | 0.600665 | 2.11E-29 |
| *Onecut2* | 0.596772 | 1.15E-36 |
| *Aplp1* | 0.592871 | 7.78E-36 |
| *Ankrd33b* | 0.589047 | 1.82E-45 |
| *Tcaf1* | 0.586252 | 3.45E-34 |
| *Rnf182* | 0.58271 | 2.56E-46 |
| *Nrep* | 0.579567 | 8.47E-29 |
| *Uchl1* | 0.578256 | 1.25E-34 |
| *Map6* | 0.577639 | 3.33E-37 |
| *Mapk8ip1* | 0.576475 | 4.83E-36 |
| *Cd59a* | 0.571505 | 1.10E-30 |
| *Otx2* | 0.570729 | 8.89E-102 |
| *Ssrp1* | 0.570481 | 4.76E-26 |
| *Cald1* | 0.569969 | 4.59E-33 |
| *Vps37b* | 0.566466 | 6.95E-32 |
| *Sox9* | 0.56189 | 3.70E-29 |
| *Rplp0* | 0.552233 | 2.43E-105 |
| *1110038B12Rik* | 0.552043 | 3.49E-35 |
| *App* | 0.54823 | 9.59E-36 |
| *Olfm1* | 0.545606 | 7.61E-32 |
| *Smpdl3a* | 0.541231 | 6.18E-36 |
| *Cd47* | 0.540681 | 4.41E-17 |
| *Xist* | 0.54059 | 0.000691 |
