# Supplementary material for: Lineage tracing reveals photoreceptor precursor cell subpopulations that contribute to murine retinogenesis
Source: Front Cell Dev Biol. 2026 Jun 4;14:1814134. doi: 10.3389/fcell.2026.1814134 (PMC13276796; doi:10.3389/fcell.2026.1814134)
Supplement: Supplementary file 13 [file Table6.docx]

**Supplemental Table S6. qPCR analysis of *Crx+* subpopulation markers in developing mouse retinas.**

qPCR analysis was performed in triplicate and ΔΔCt analysis was performed using E14 and *B2m* as references.

| Gene | E14 | E16 | P0 | P3 | P4 | P5 | P6 | P15 | P30 |
| --- | --- | --- | --- | --- | --- | --- | --- | --- | --- |
| *B2m* | 1.0084 | 1.0043 | 1.0087 | 1.0144 | 1.0003 | 1.0078 | 1.0087 | 1.0181 | 1.0360 |
| *Crx* | 1.0267 | 1.0769 | 2.1259 | 15.5637 | 24.9475 | 16.7941 | 22.3823 | 9.9888 | 18.4700 |
| *Dll1* | 1.0123 | 0.6770 | 0.8319 | 1.0349 | 0.9155 | 0.6641 | 0.5494 | 0.1643 | 0.1976 |
| *Cd24a* | 1.0224 | 0.6800 | 0.4884 | 0.6229 | 0.6177 | 0.5590 | 0.3802 | 0.0304 | 0.0216 |
| *Rlbp1* | 1.0256 | 1.3104 | 4.4002 | 53.4225 | 66.2308 | 58.6600 | 65.0563 | 67.6127 | 82.2933 |
| *Crym* | 1.0150 | 1.9863 | 4.7870 | 23.2967 | 26.7308 | 29.1513 | 32.1317 | 3.5600 | 3.6453 |
| *Pon2* | 1.0353 | 1.0929 | 1.3023 | 2.7226 | 2.2186 | 1.5590 | 1.6924 | 2.7806 | 1.8150 |
| *Tspan15* | 1.0046 | 0.8496 | 1.0733 | 1.5261 | 1.4675 | 1.1515 | 0.8833 | 0.2128 | 0.2088 |
| *Cnp* | 1.0310 | 1.2582 | 2.0243 | 6.6012 | 6.3974 | 4.7660 | 4.7499 | 0.8861 | 2.9463 |
| *Neurod4* | 1.0184 | 0.9738 | 1.4074 | 7.2610 | 6.7807 | 5.8128 | 3.6054 | 2.7582 | 2.7016 |
| *Trib2* | 1.0046 | 0.8657 | 0.9203 | 3.3451 | 3.7174 | 3.0460 | 3.9024 | 1.1274 | 1.8538 |
| *Igfbp2* | 1.0282 | 0.5996 | 0.2591 | 0.5189 | 0.4392 | 0.3389 | 0.5386 | 0.0865 | 0.0912 |
| *Cmtm7* | 1.0261 | 1.2277 | 1.1131 | 2.1403 | 1.3124 | 0.7881 | 0.6379 | 0.0833 | 0.0700 |
| *Dll3* | 1.0092 | 1.0043 | 0.7534 | 0.9838 | 0.8671 | 0.4929 | 0.3522 | 0.0075 | 0.0091 |
| *Cadm3* | 1.0093 | 1.0867 | 0.8862 | 3.1451 | 4.1637 | 3.7791 | 3.0881 | 3.7629 | 3.1046 |
| *Abca4* | 1.0032 | 0.5129 | 1.6547 | 61.9336 | 107.0055 | 109.5007 | 169.8574 | 967.2584 | 893.6749 |
| *Prom1* | 1.0170 | 0.8389 | 0.8311 | 5.5532 | 10.1884 | 7.3248 | 8.7523 | 11.4721 | 11.4348 |
| *Nt5e* | 1.0025 | 0.7396 | 4.2015 | 67.8343 | 118.6630 | 99.6982 | 97.5510 | 137.3660 | 163.8082 |
| *Pias3* | 1.0011 | 0.6989 | 2.1137 | 10.8064 | 17.3083 | 17.5273 | 16.2146 | 5.3154 | 8.1630 |
| *Nrl* | 1.0898 | 2.1544 | 19.7699 | 392.7972 | 820.0937 | 645.3190 | 755.8938 | 1186.2757 | 1639.4028 |
